# Tobacco smoking and risk of 36 cardiovascular disease subtypes: fatal and non-fatal outcomes in a large prospective Australian study

**DOI:** 10.1186/s12916-019-1351-4

**Published:** 2019-07-03

**Authors:** Emily Banks, Grace Joshy, Rosemary J. Korda, Bill Stavreski, Kay Soga, Sam Egger, Cathy Day, Naomi E. Clarke, Sarah Lewington, Alan D. Lopez

**Affiliations:** 1 National Centre for Epidemiology and Population Health, Research School of Population Health, Australian National University, Mills Road, Acton, ACT 2601 Australia; 2 The Sax Institute, Sydney, Australia; 3 National Heart Foundation of Australia, Melbourne, Australia; 4 Cancer Council NSW, Sydney, Australia; 5 Clinical Trials Service Unit, Nuffield Department of Population Health, University of Oxford, Oxford, UK; 6 Medical Research Council Population Health Research Unit, Nuffield Department of Population Health, University of Oxford, Oxford, UK; 7 Melbourne School of Population and Global Health, University of Melbourne, Melbourne, Australia

**Keywords:** Cardiovascular disease, Smoking, Tobacco, Ischaemic heart disease, Coronary heart disease, Cerebrovascular disease, Arrhythmia, Atrial fibrillation, Heart failure, Cardiovascular mortality

## Abstract

**Background:**

Tobacco smoking is a leading cause of cardiovascular disease (CVD) morbidity and mortality. Evidence on the relation of smoking to different subtypes of CVD, across fatal and non-fatal outcomes, is limited.

**Methods:**

A prospective study of 188,167 CVD- and cancer-free individuals aged ≥ 45 years from the Australian general population joining the 45 and Up Study from 2006 to 2009, with linked questionnaire, hospitalisation and death data up to the end of 2015. Hazard ratios (HRs) for hospitalisation with or mortality from CVD among current and past versus never smokers were estimated, including according to intensity and recency of smoking, using Cox regression, adjusting for age, sex, urban/rural residence, alcohol consumption, income and education. Population-attributable fractions were estimated.

**Results:**

During a mean 7.2 years follow-up (1.35 million person-years), 27,511 (crude rate 20.4/1000 person-years) incident fatal and non-fatal major CVD events occurred, including 4548 (3.2) acute myocardial infarction (AMI), 3991 (2.8) cerebrovascular disease, 3874 (2.7) heart failure and 2311 (1.6) peripheral arterial disease (PAD) events. At baseline, 8% of participants were current and 34% were past smokers. Of the 36 most common specific CVD subtypes, event rates for 29 were increased significantly in current smokers. Adjusted HRs in current versus never smokers were as follows: 1.63 (95%CI 1.56–1.71) for any major CVD, 2.45 (2.22–2.70) for AMI, 2.16 (1.93–2.42) for cerebrovascular disease, 2.23 (1.96–2.53) for heart failure, 5.06 (4.47–5.74) for PAD, 1.50 (1.24–1.80) for paroxysmal tachycardia, 1.31 (1.20–1.44) for atrial fibrillation/flutter, 1.41 (1.17–1.70) for pulmonary embolism, 2.79 (2.04–3.80) for AMI mortality, 2.26 (1.65–3.10) for cerebrovascular disease mortality and 2.75 (2.37–3.19) for total CVD mortality. CVD risks were elevated at almost all levels of current smoking intensity examined and increased with smoking intensity, with HRs for total CVD mortality in current versus never smokers of 1.92 (1.11–3.32) and 4.90 (3.79–6.34) for 4–6 and ≥ 25 cigarettes/day, respectively. Risks diminished with quitting, with excess risks largely avoided by quitting before age 45. Over one third of CVD deaths and one quarter of acute coronary syndrome hospitalisations in Australia aged < 65 can be attributed to smoking.

**Conclusions:**

Current smoking increases the risk of virtually all CVD subtypes, at least doubling the risk of many, including AMI, cerebrovascular disease and heart failure. Paroxysmal tachycardia is a newly identified smoking-related risk. Where comparisons are possible, smoking-associated relative risks for fatal and non-fatal outcomes are similar. Quitting reduces the risk substantially. In an established smoking epidemic, with declining and low current smoking prevalence, smoking accounts for a substantial proportion of premature CVD events.

**Electronic supplementary material:**

The online version of this article (10.1186/s12916-019-1351-4) contains supplementary material, which is available to authorized users.

## Introduction

Cardiovascular disease (CVD) is the leading cause of mortality globally and encompasses a broad and diverse range of subtypes, including ischaemic heart disease (IHD), cardiac dysrhythmias, cerebrovascular disease, peripheral arterial disease and heart failure [1].

Smoking increases the risk of CVD [2–6]. However, the magnitude of this increase in risk varies substantively according to a range of factors. Smoking-related CVD risks are highest in current and recent smokers, compared to never smokers and those who have quit in the more distant past [5]. Risk also increases with increasing duration of use and with greater intensity of smoking, as measured by the number of cigarettes smoked per day [5]. The data that are available indicate variation according to CVD subtype [5–7]. For example, while mortality from both IHD and abdominal aortic aneurysm is elevated in current smokers, compared to those who have never smoked, contemporary US cohort study data show considerable variation in the relative risks, from 2.6 (95%CI 2.4–2.7) for IHD mortality to 7.5 (5.8–9.7) for mortality from abdominal aortic aneurysm for men [6]. In general, studies to date have focused on fatal CVD outcomes, with limited evidence on non-fatal outcomes. As with most risk factors for CVD, excess relative risks attributable to current smoking tend to decline with age [5], and absolute excess risks increase [8]. While data on variation in risk according to other factors are limited, there is some evidence of a greater impact of smoking on IHD in women than in men [9]. Relative and absolute excess CVD risks attributable to smoking will vary according to the absolute CVD risk in never smokers [8].

Hence, smoking-attributable risks vary according to the CVD outcome under examination and according to the pattern of smoking in a population, which, in turn, relate to the stage of the tobacco epidemic and will vary over time and between countries [10]. It follows that a comprehensive contemporary picture of the relationship of smoking to CVD, necessary for appropriate policy and practice responses, requires examination of the full spectrum of CVD, including fatal and non-fatal outcomes, according to smoking intensity and recency. However, our search of the published evidence to date did not locate any studies that included all of these features (see the “Discussion” section and Additional file 1: Brief systematic review of smoking and cardiovascular disease). Moreover, estimates of the proportion of CVD events attributable to smoking in the population often present summaries for the whole population [11], which tend to be weighted towards older ages, rather than providing age-specific evidence and evidence relating to premature CVD, which is more informative for tobacco control.

This study aimed to quantify the relation of tobacco smoking, including measures of intensity and recency, to the risk of CVD and CVD subtypes, including acute myocardial infarction, cerebrovascular disease, heart failure, peripheral arterial disease and dysrhythmias, considering fatal and non-fatal outcomes, where appropriate. The relationship of smoking to outcomes in different population subgroups was also considered, along with the proportion and number of CVD deaths attributable to smoking, by age.

In common with North America and much of Europe, Australia is considered to have a mature smoking epidemic, with historically high prevalences of smoking, high intensity and very long durations of smoking among smokers in the population, similar prevalences of smoking among men and women and low and decreasing current prevalence of smoking [10]. The findings of this study are therefore likely to be informative internationally, as well as nationally.

## Methods

The Sax Institute’s 45 and Up Study is a cohort study of 267,153 men and women aged 45 and over at baseline, randomly sampled from the general population of New South Wales (NSW), Australia, using the Department of Human Services enrolment database. Individuals joined the study by completing a postal questionnaire from January 2006 to December 2009 and are followed up through repeated data collection and through data linkage. The general study methods are described in detail elsewhere [12].

Baseline questionnaire data include information on socio-demographic factors, health behaviours, height and body weight, medical and surgical history, functional capacity and physical activity. The study questionnaire is available at https://www.saxinstitute.org.au/our-work/45-up-study/questionnaires/.

Questionnaire data from study participants were linked probabilistically to the data from the NSW Admitted Patient Data Collection, which is a complete census of all public and private hospital admissions in NSW, the NSW Register of Births, Deaths and Marriages (to provide fact of death); the Australian Bureau of Statistics Cause of Death unit record files (to provide cause of death); and the National Death Index (to provide fact and cause of death), up to the end of December 2015. Combined, these death registration data capture all deaths in New South Wales and Australia. The linked Admitted Patient Data Collection data that were used contained details of admissions in participants from the year 2001 up to the end of December 2015, including the primary reason for admission using the International Classification of Diseases 10th revision–Australian Modification (ICD-10-AM) [13] and up to 50 additional clinical diagnoses. Probabilistic linkage is conducted by the NSW Centre for Health Record Linkage and is known to be highly accurate (false-positive and false-negative rates < 0.4%) [14].

### Statistical methods

Participants with the following were excluded: study withdrawal since baseline, invalid data on age or date of recruitment (*n* = 461, 0.17%), data linkage errors (*n* = 195, 0.07%) and missing or invalid data on smoking status (*n* = 851, 0.32%). To minimise the potential impact of reverse causality or the “sick quitter” effect, participants with a history of CVD or cancer at baseline were excluded. A history of CVD at baseline was defined as self-reported heart disease, stroke or blood clot on the baseline questionnaire, or a hospital admission in the 5 years prior to entering the study with a major CVD diagnosis code [15] in any diagnostic field or a CVD-related procedure code in any procedure code field (*n* = 56,891, 21.3%). A 5-year window was used to ensure a uniform probability of identification of previous diagnoses from administrative data for all participants. Cancer was defined as having a self-reported history of doctor-diagnosed cancer other than melanoma and non-melanoma skin cancer (*n* = 20,588, 7.7%). Our analysis dataset consisted of 188,167 participants. Current smokers with missing data on smoking intensity (*n* = 309) and past smokers with missing data on age at quitting smoking (*n* = 3071) were excluded from the analyses using those variables.

Smoking status was classified according to the responses to the following series of items on the baseline questionnaire, as applied previously [16]: “Have you ever been a regular smoker?” If “Yes”, “How old were you when you started smoking regularly?” “Are you a regular smoker now?” If “No”, “How old were you when you stopped smoking regularly?” “About how much do you/did you smoke on average each day?” Never smokers were participants who answered “No” to the first question, “Have you ever been a regular smoker?” Current smokers were those who answered “Yes” to the first question and also “Yes” to the question about being a smoker now, and past smokers were those who indicated that they had ever been a regular smoker and that they were not a regular smoker now. The question on the number of cigarettes, pipes or cigars smoked per day was used to categorise the smoking intensity of current smokers as 1–14, 15–24 and ≥ 25 cigarettes/pipes/cigars day (referred to as “cigarettes/day” due to the small number of current smokers using other tobacco types (*n* = 381)).

Identification of incident CVD outcomes was based on all 51 diagnosis code fields in the hospital data and the underlying cause of death codes in the death data. The primary outcomes were a combined endpoint of hospitalisation with or mortality attributed to a specific CVD cause. We also reported on fatal outcomes alone to allow investigation of this outcome and comparison with international findings. Incident (fatal or non-fatal) CVD outcomes included (i) a composite outcome of “major CVD”, which included serious atherosclerotic and thromboembolic disease within the ICD-10-AM circulatory disease chapter, excluding less life-threatening circulatory conditions such as haemorrhoids and varicose veins [15]; (ii) common CVD subgroups, such as ischaemic heart disease (IHD, ICD-10-AM I20-I25) as well as acute myocardial infarction separately (AMI, I21), heart failure (I50), cerebrovascular disease (I61-I67, I69), and peripheral arterial disease (ICD-10-AM I70-I74); and (iii) specific CVD subtypes with at least 50 events, defined by individual level 3 ICD-10-AM codes included in the definition of major CVD, such as atrial fibrillation, cardiomyopathy, pulmonary embolism and abdominal aortic aneurysm, as well as for essential (primary) hypertension (I10) and vascular disorders of intestine (K55) [5, 6]. In analyses defined post hoc, additional categories of paroxysmal tachycardia (I47) – supraventricular tachycardia (I47.1) and ventricular tachycardia (I47.2) – were investigated.

For each CVD outcome, eligible participants contributed person-years from the date of recruitment until the first occurrence of the outcome, date of death or end of follow-up (31 December 2015), whichever was the earliest. Incidence rates and 95% confidence intervals (95%CI) were calculated separately for participants who reported being current, past or never smokers at baseline; the sex-specific rates were age-standardised to the 2006 NSW population, in 5-year age groups, using the direct method [17]; rates for males and females combined were age- and sex-standardised.

Hazard ratios (HRs) were used to approximate relative risks (RRs) for CVD outcomes and were estimated for current and past smokers, compared to never smokers, using Cox regression modelling, with age as the underlying time variable. RRs for CVD mortality were estimated separately, where data were sufficient. Separate regression models were fitted for each CVD outcome, allowing risk sets to be outcome specific; for example, someone experiencing a non-fatal IHD event would continue to be in the risk set for other non-fatal CVD outcomes. Models are adjusted for covariates selected a priori and derived from baseline questionnaire and participant location data including age (automatically adjusted for as the underlying time variable), sex, education [no school certificate, certificate/diploma/trade, university degree], annual pre-tax household income [AUD < $20,000; $20,000–$39,999; $40,000–$69,999; ≥ $70,000], region of residence [major cities, inner regional, outer regional/remote/very remote] and alcohol consumption [0, 1–14, ≥ 15 alcoholic drinks/week]. Additional adjustment for region of birth did not materially change the point estimates (< 5%), so the most parsimonious model was retained. Factors considered likely to contribute, even partially, to the causal pathway between smoking and CVD—including body mass index (and related factors of diabetes and treatment for high cholesterol), high blood pressure and physical activity—were not adjusted for. Instead, these were considered in the subgroup analyses. Sensitivity analyses estimated RRs for incident major CVD by smoking status, excluding the first year of follow-up and, separately, re-categorising current smokers to include past smokers who had ceased smoking 3 or fewer years prior to baseline.

In addition, RRs for incident CVD outcomes were estimated for current smokers according to the number of cigarettes smoked per day (categorised as 1–14, 15–24 or ≥ 25 cigarettes/day), compared to never smokers; analyses were then conducted with finer categories of smoking intensity (including 1–3, 4–6 and 7–9 cigarettes/day) for any major CVD outcomes. RRs were plotted by smoking intensity, against the mean number of cigarettes within each category reported at a 3-year resurvey completed by a sample of participants, among those who reported being current smokers at resurvey [16]. This resurvey result was considered to be the best estimate of long-term mean consumption among all in a particular category, before the study started. The RRs for incident CVD outcomes were also quantified among past smokers versus never smokers overall, and in those ceasing smoking at ages < 25, 25–34, 35–44 and 45–54 years versus never smokers, excluding past smokers quitting at age 55 or older (*n* = 7485).

The RRs for acute myocardial infarction by smoking status were also estimated within subgroups defined according to the following factors and tests for statistical interaction conducted: age (45–64, 65–79 and ≥ 80 years), sex, alcohol intake, education, income, private health insurance, physical activity tertiles weighted for intensity, region of residence, doctor-diagnosed diabetes, physical functional limitation (Medical Outcomes Study–Physical Functioning scale [18]), body mass index, treatment for high blood pressure, treatment for high cholesterol, aspirin use and country of birth. This outcome was chosen as it is common and important, with excellent capture using hospitalisation and mortality data. Variation in the relationship of current and past smoking to broadly grouped CVD outcomes according to sex was also examined.

The proportionality assumptions were verified using tests based on Schoenfeld residuals. A stratified form of the model was used where covariates displayed non-proportionality of hazards. Tests for trend were performed by modelling the exposure categories of smoking intensity, mean number of cigarettes smoked, as an ordinal variable. Likelihood ratio tests were used to assess interaction with smoking status; models using time since baseline as the timescale and adjusted for age (5-year age groups) were used to assess interaction with age. All statistical tests were two-sided, using a significance level of 5%, except for the tests based on Schoenfeld residuals, where a significance level of 0.00001 was used due to the large sample size. In all Cox regression modelling, missing values of covariates were included in the models as separate categories while cases with missing values of main exposures (smoking status, smoking intensity and age at smoking cessation) were excluded.

Finally, we calculated the smoking-attributable fraction (SAF) according to sex and age group for CVD mortality and for hospitalisation for acute coronary syndrome. We chose these outcomes because national data on their occurrence were available for Australia. We used the prevalence-based method, as per the following formula:

$$ \mathrm{SAF}\left(\%\right)=\frac{100\times \left[{p}_{\mathrm{fs}}\left(\mathrm{R}{\mathrm{R}}_{\mathrm{fs}}-1\right)+{p}_{\mathrm{cs}}\left(\mathrm{R}{\mathrm{R}}_{\mathrm{cs}}-1\right)\right]}{\left[{p}_{\mathrm{fs}}\left(\mathrm{R}{\mathrm{R}}_{\mathrm{fs}}-1\right)+{p}_{\mathrm{cs}}\left(\mathrm{R}{\mathrm{R}}_{\mathrm{cs}}-1\right)+1\right]} $$

where *p*_fs_ and *p*_cs_ represent the prevalence of former and current smoking, respectively, and RR_fs_ and RR_cs_ represent the hazard ratios for outcomes among former and current smokers, respectively, relative to never smokers. For the estimates of the likely contemporary and near-future SAFs, we used estimates of smoking prevalence from the 2014–2015 Australian National Health Survey [19]. For the calculation of the SAF for events occurring within the cohort during follow-up, estimates of smoking prevalence from the 2004–2005 Australian National Health Survey were used in supplementary analyses [20], to allow for an aetiologically relevant lag time between the exposure and outcome [21, 22]. Age group-specific RRs (45–74 years and ≥ 75 years) from models with age as the underlying time variable, adjusting for sex, region of residence, alcohol consumption, annual household income and education attainment, were used due to the heterogeneity across the subgroups of age. Observed mortality for circulatory disease in the Australian population [23] and our estimates of SAFs for mortality from major CVD were used to calculate the population-attributable fractions and the number of smoking-attributable CVD deaths. Estimates of age- and sex-specific SAFs for AMI, applied to observed hospitalisations for unstable angina and AMI in the Australian population [24], were used to calculate the population-attributable fractions and the number of smoking-attributable hospitalisations for these outcomes.

## Results

At baseline, 8% of the 188,167 participants reported being current smokers and 34% were past smokers. The prevalence of smoking was similar in men and women. Overall, 94.9% (*n* = 74,141) of all current and past smokers reported smoking cigarettes only, 1.8% (*n* = 1379) smoked cigarettes and cigars or pipes and 1.8% (*n* = 1407) smoked only cigars or pipes.

Compared to never smokers, current smokers were, on average, younger, less likely to be urban residents, of lower income and education level, and less likely to hold private health insurance; they were more likely to report consuming ≥ 15 alcoholic drinks/week and to have a body mass index < 20 kg/m^2^ (Table 1).

**Table 1:** Characteristics of study participants at baseline by smoking status

	Current			Past	Never
	≥ 15 cigarettes/day	< 15 cigarettes/day	Total		
Total	5% (9400)	3% (5002)	8% (14,711)	34% (63,427)	58% (110,029)
Age					
45–64 years	85% (8027)	83% (4175)	84% (12,425)	69% (43,722)	70% (77,546)
65–79 years	14% (1269)	14% (694)	14% (2024)	25% (15,982)	23% (25,449)
≥ 80 years	1% (104)	3% (133)	2% (262)	6% (3723)	6% (7034)
Men	51% (4792)	43% (2175)	48% (7120)	53% (33,548)	38% (42,124)
Residing in major cities	48% (4408)	51% (2533)	49% (7099)	51% (31,783)	54% (58,685)
University degree	12% (1114)	19% (946)	15% (2109)	23% (14,378)	29% (31,151)
Household income ≥ $70,000	21% (1578)	26% (1051)	23% (2673)	34% (17,322)	37% (31,869)
Private health insurance (hospital/DVA)	39% (3700)	50% (2502)	43% (6329)	65% (41,254)	71% (78,049)
≥ 15 alcoholic drinks per week	28% (2528)	20% (982)	25% (3561)	23% (14,540)	9% (9164)
Highest physical activity tertile	35% (3134)	37% (1783)	35% (5008)	38% (23,745)	35% (36,901)
Physical functioning limitation (MOS-PF < 75)	23% (1886)	16% (694)	21% (2632)	15% (8371)	13% (12,510)
Body mass index					
15 to 19.9 kg/m^2^	7% (578)	7% (344)	7% (947)	3% (1686)	4% (4361)
30 to 50 kg/m^2^	22% (1929)	18% (823)	21% (2812)	24% (14,417)	20% (20,431)
Doctor diagnosed diabetes	7% (677)	5% (266)	7% (969)	8% (4975)	6% (6790)
Treatment for high blood pressure	17% (1552)	13% (657)	15% (2259)	21% (13,566)	21% (22,717)
Treatment for high cholesterol	11% (1034)	9% (470)	10% (1537)	13% (8540)	12% (12,927)
Taking aspirin	12% (1164)	12% (585)	12% (1786)	16% (9850)	13% (14,769)

Percentages shown in the “Total” row represent proportions of the total study population (*n* = 188,167). All other percentages represent proportions within smoking status categories. Denominators of the percentages do not include missing data. Number of study participants with missing data: region of residence = 3637; education= 2749; annual household income = 39,060; private health insurance = 5; alcohol consumption = 3606; physical activity tertile = 5713; physical functioning limitation = 22,978; body mass index = 13,753; taking aspirin = 18; smoking intensity = 309; and other variables = 0

*DVA* Department of Veterans’ Affairs, *MOS-PF* Medical Outcomes Study–Physical Functioning scale

During a total follow-up time of 1.35 million person-years (mean 7.4 years), there were 27,511 major CVD events—fatal and non-fatal. These included the following: 11,778 IHD, including 4548 AMI; 3991 cerebrovascular disease; 3874 heart failure; and 2311 peripheral arterial disease events. There were 2540 deaths from major CVD: 1101 from IHD including 558 from AMI, 697 from cerebrovascular disease and 135 from peripheral arterial disease.

**Fig. 1:**
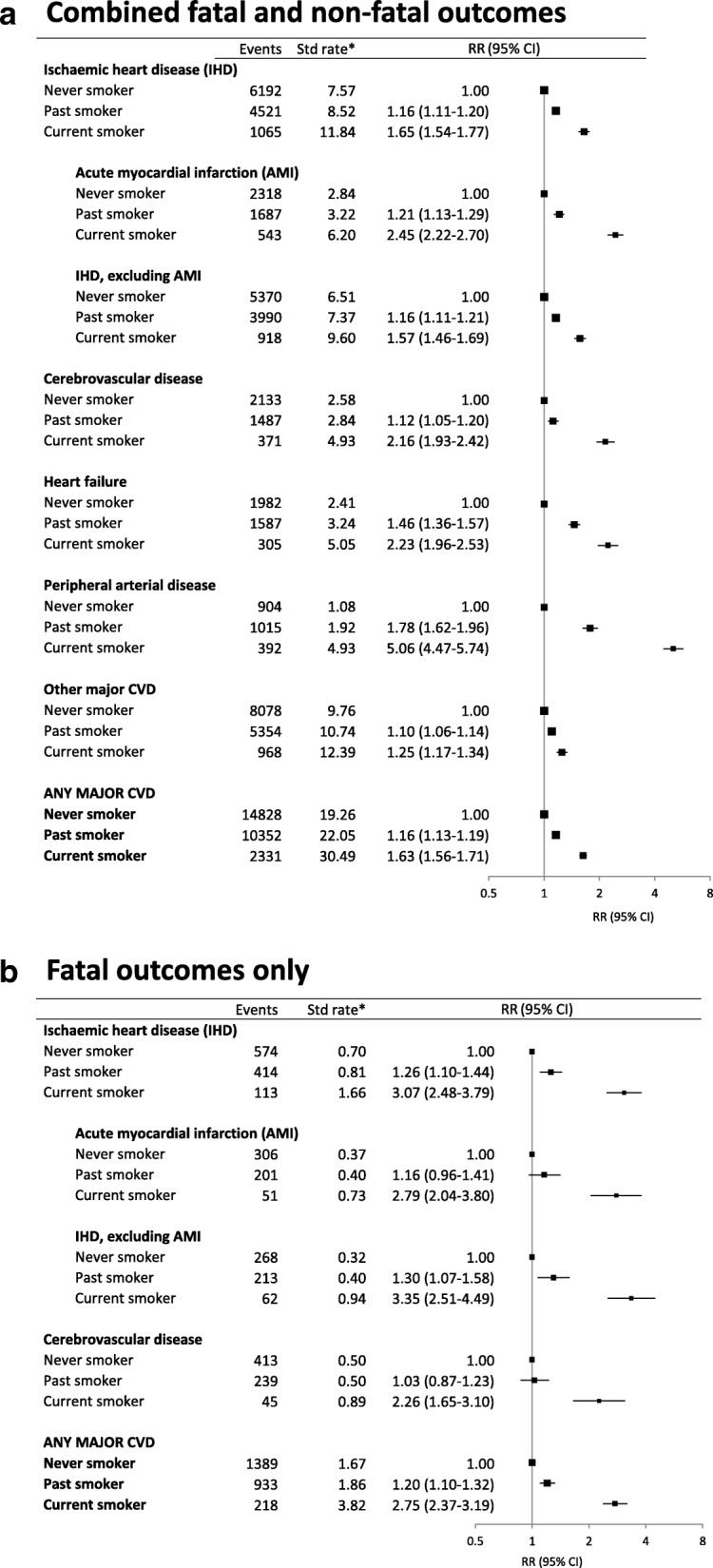
Grouped CVD subtype outcomes in relation to smoking status at baseline. **a** Combined fatal and non-fatal outcomes. **b** Fatal outcomes only. *Age-sex-standardised rate per 1000 person-years. RR (relative risk) adjusted for age, sex, region of residence, alcohol consumption, annual household income and education attainment; adjustment for sex is through stratification for outcomes of IHD, AMI, non-AMI, IHD, heart failure (stratified for income as well) and major CVD in **a** and for IHD and major CVD in **b**. They are plotted on a log scale and are represented by squares of areas proportional to the natural logarithm of the number of events, indicating the amount of statistical information available. Ischaemic heart disease (IHD) (ICD-10-AM codes I20–I25); acute myocardial infarction (I21); IHD, excluding AMI (I20, I22–I25); cerebrovascular disease (I61–I67 and I69); heart failure (I50); peripheral arterial disease (I70–I74); other major CVD (any major CVD except ischaemic heart disease, cerebrovascular disease, heart failure, and peripheral arterial disease); and any major CVD (as defined in reference [15]). Note that the number of events in subtypes will not add up to the total events in any major CVD as the subtypes are not mutually exclusive

Rates of hospitalisation with or death from acute myocardial infarction, cerebrovascular disease, heart failure, peripheral arterial disease and major CVD combined were elevated in current versus never smokers, for men and women (Fig. 1a, b; Additional file 2: Table S1). Former smokers showed an intermediate level of risk. Compared to never smokers, current smokers were at a significantly increased risk of hospitalisation with or mortality from IHD (adjusted RR 1.65, 95%CI 1.54–1.77), including AMI (2.45, 2.22–2.70); cerebrovascular disease (2.16, 1.93–2.42); heart failure (2.23, 1.96–2.53); peripheral arterial disease (5.06, 4.47–5.74); and any major CVD (1.63, 1.56–1.71) (Fig. 1a). These estimates did not change materially when the first year of follow-up was excluded or when recent quitters were re-classified as current smokers (Additional file 2: Tables S2 and S3). The RRs of non-fatal events for these outcomes are given in Additional file 2: Table S4. The RR of dying from AMI in current versus never smokers was 2.79 (2.04–3.80); the corresponding result for mortality from cerebrovascular disease was 2.26 (1.65–3.10) (Fig. 1b). The RR of dying from any major CVD in current versus never smokers was 2.75 (2.37–3.19) (Fig. 1b).

**Fig. 2:**
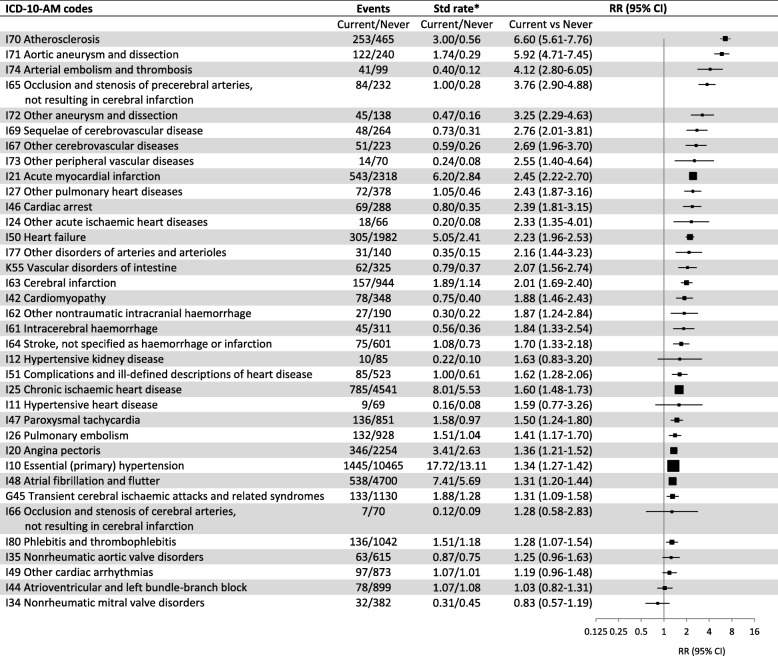
Combined fatal and non-fatal outcomes for specific CVD subtypes (level 3 ICD-10-AM codes) with at least 50 events, in current versus never smokers. *Age-sex-standardised rate per 1000 person-years. RR (relative risk) adjusted for age, sex, region of residence, alcohol consumption, annual household income and education attainment; adjustment for sex is through stratification for outcomes I21, I25, I48, I50 (stratified for income as well) and I10 (stratified for alcohol consumption and income as well). I10 and K55 are not part of “any major CVD” but were included in this table due to their relevance as outcomes related to smoking, see Fig. 1 for details of RR plots

When considered in finer pathological and clinical categories (single level 3 ICD-10-AM codes), significantly elevated risks in current versus never smokers were observed for 29 of the 36 subtypes investigated (Fig. 2). Details of the finer CVD subtypes contributing to the numbers of fatal and non-fatal events, separately, and age-standardised rates of disease according to smoking status are given in Additional file 2: Tables S5 and S6. The markedly elevated RRs for current versus never smokers of combined fatal and non-fatal outcomes relating to peripheral arterial disease were observed for subcategories of that disease, including for abdominal aortic aneurysm (ICD-10-AM I71) and atherosclerosis (I70) (Fig. 2). Significantly increased risks of hospitalisation/mortality for vascular disorders of the intestine (K55) and subcategories of cerebrovascular disease and IHD were observed, although the latter showed significant heterogeneity, with higher RR for AMI (I21) and lesser elevations in risk for chronic ischaemic heart disease (I25) and angina (I20). Compared to people who had never smoked, current smokers had significantly elevated risks of heart failure (I50); cardiomyopathy (I42); venous thromboembolism, including pulmonary embolism (I26) and deep vein thrombosis (I80); dysrhythmias, including atrial fibrillation and flutter (I48) and paroxysmal tachycardia (I47); and essential hypertension (I10). The RR of supraventricular tachycardia (I47.1) in current versus never smokers was 1.35 (1.07-1.72) and that of ventricular tachycardia (I47.2) was 1.82 (1.35-2.42). Combined hospitalisation/mortality outcomes for various valvular disorders (I34, I35) and atrioventricular and left bundle-branch block and other cardiac arrhythmias (I44, I49) did not show significant associations with current smoking (Fig. 2).

**Fig. 3:**
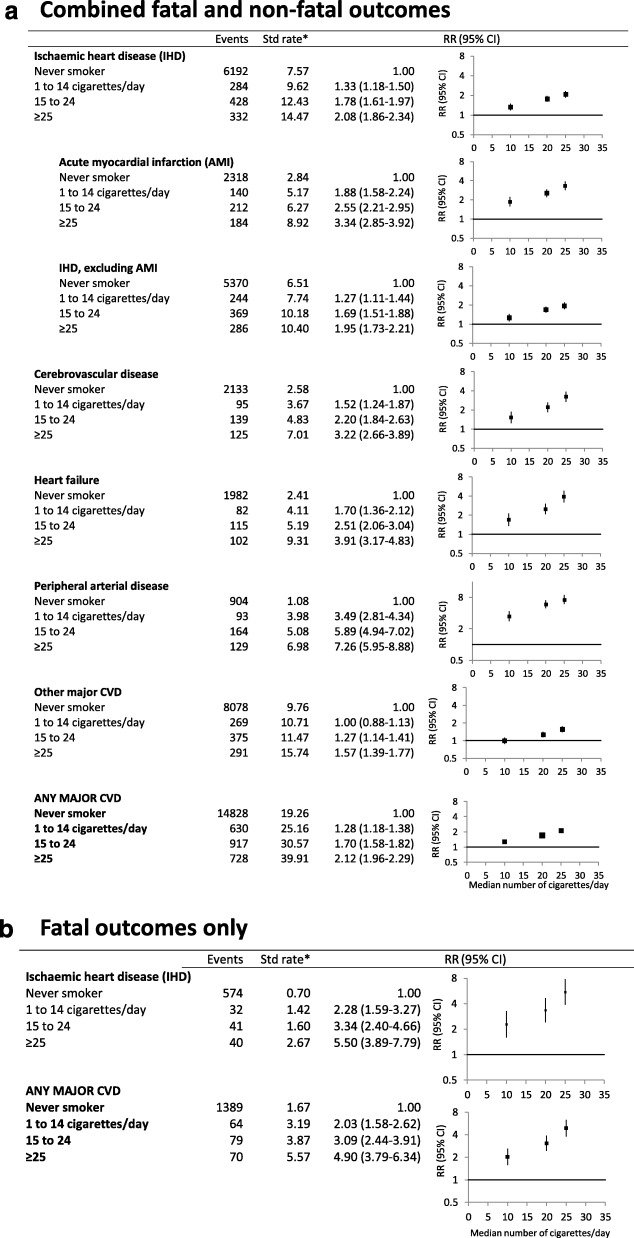
Grouped CVD subtype outcomes for current smokers, by smoking intensity, compared to never smokers at baseline. **a** Combined fatal and non-fatal outcomes. **b** Fatal outcomes only. *Age-sex-standardised rate per 1000 person-years. RR (relative risk) adjusted for age, sex, region of residence, alcohol consumption, annual household income and education attainment; adjustment for sex is through stratification for outcomes of IHD, AMI, non-AMI IHD and major CVD in **a**. *p*_trend_ < 0.0001 for all outcomes. Categories of smoking intensity (never smokers, 1 to 14 cigarettes/day, 15 to 24, and ≥ 25) are based on smoking behaviour reported at baseline. RRs are plotted on a log scale against the median number of cigarettes per day within each of the pre-defined categories, based on smoking intensity reported at follow-up among current smokers. Note different scales used for *y*-axis, see Fig. 1 for details of RR plots and outcome definitions

**Fig. 4:**
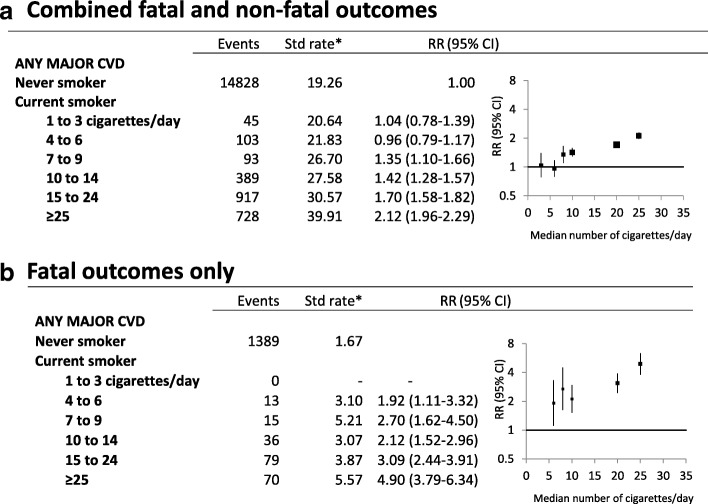
Major CVD outcomes in current smokers, by fine categories of smoking intensity, compared to never smokers at baseline. **a** Combined fatal and non-fatal outcomes. **b** Fatal outcomes only. *Age-sex-standardised rate per 1000 person-years. RR (relative risk) adjusted for age, sex, region of residence, alcohol consumption, annual household income and education attainment; adjustment for sex is through stratification for major CVD in **a**. *p*_trend_ < 0.0001 for all outcomes. Categories of smoking intensity (never smokers, 1 to 3 cigarettes/day, 4 to 6, 7 to 9, 10 to 14, 15 to 24, and ≥ 25) are based on smoking behaviour reported at baseline. RRs are plotted on a log scale against the median number of cigarettes per day within each of the pre-defined categories, based on smoking intensity reported at follow-up among current smokers, see Fig. 1 for details of RR plots and outcome definitions

The RRs for the main CVD outcomes increased with increasing intensity of smoking, among current smokers relative to never smokers (Fig. 3a, b; *p*_trend_ < 0.0001 for all outcomes). There was a doubling in the rate of major CVD events (fatal and non-fatal) in current smokers of 25 or more cigarettes per day, compared with never smokers (2.12, 1.96–2.29); at least a tripling in the rate of AMI (3.34, 2.85–3.92), cerebrovascular disease (3.22, 2.66–3.89) or heart failure (3.91, 3.17–4.83); and a roughly sevenfold risk of peripheral arterial disease (7.26, 5.95–8.88). In current smokers of 25 or more cigarettes per day, compared with never smokers, RRs were 4.90 (3.79–6.34) for mortality from major CVD and 5.50 (3.89–7.79) for mortality from IHD (Fig. 3b). Considering finer increments in smoking intensity, the risk of any fatal or non-fatal major CVD was significantly elevated from a current smoking level of 7–9 cigarettes per day (1.35, 1.10–1.66) and of CVD mortality from 4 to 6 cigarettes per day (1.92, 1.11–3.32), compared to never smokers (Fig. 4a, b; *p*_trend_ < 0.0001 for both).

**Fig. 5:**
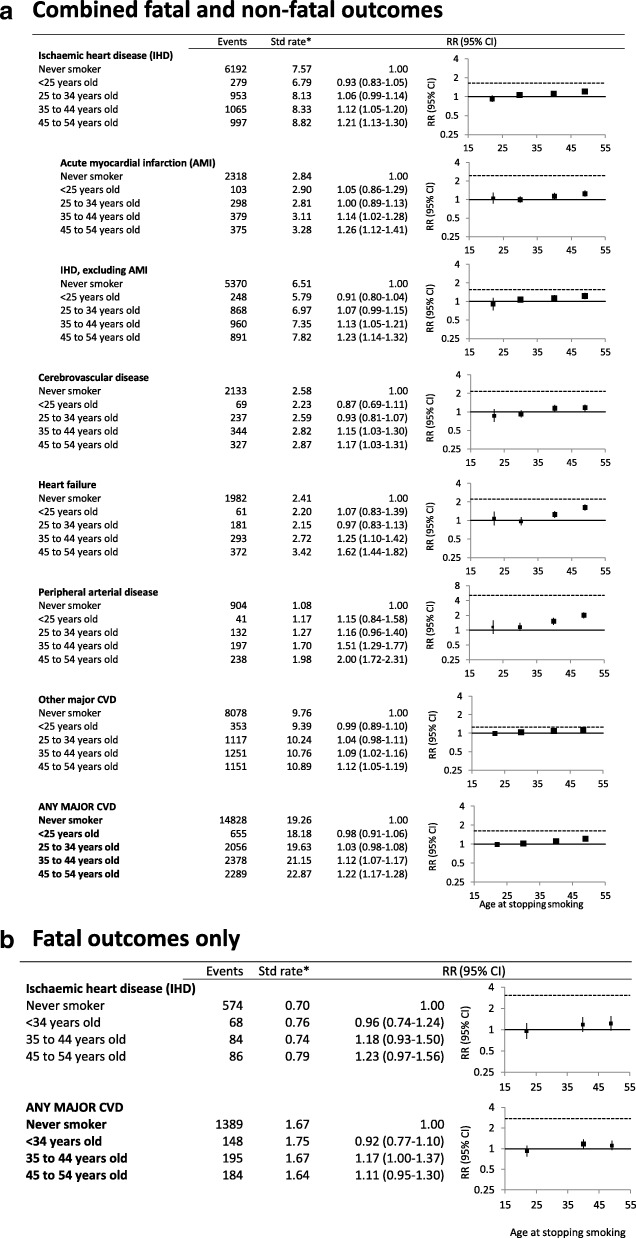
Grouped CVD subtype outcomes by age at smoking cessation, among past smokers, compared to never smokers. **a** Combined fatal and non-fatal outcomes. **b** Fatal outcomes only. *Age-sex-standardised rate per 1000 person-years. RR (relative risk) adjusted for age, sex, region of residence, alcohol consumption, annual household income and education attainment; adjustment for sex is through stratification for outcomes of IHD, AMI, non-AMI IHD, heart failure and major CVD in **a** and IHD, non-AMI IHD and major CVD in **b**. They are plotted on a log scale against median values of age categories for stopping smoking (< 25 years old, 25 to 34, 35 to 44, 45 to 54). Dotted lines above the reference lines show RRs for current smokers relative to never smokers. Note different scales used for *y*-axis, see Fig. 1 for details of RR plots and outcome definitions

For the common CVD subtypes, the risks for past smokers quitting prior to the age of 35 did not differ significantly from those of never smokers (Fig. 5a, b). Persistent elevations in the risk of peripheral arterial disease following cessation of smoking were observed, with a small elevation in the composite major CVD outcome among those quitting at age 35–44 or 45–54 years. No significant elevations in mortality from IHD and from all major CVD were seen in past smokers quitting by age 55, compared to never smokers (Fig. 5b).

**Fig. 6:**
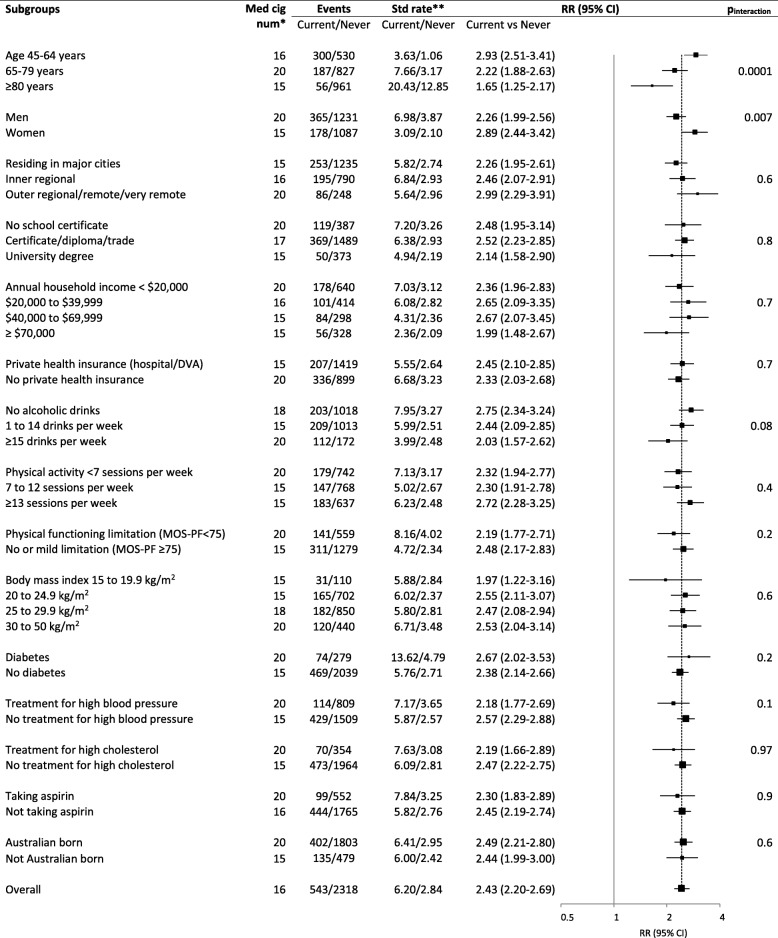
Combined fatal and non-fatal acute myocardial infarction in current smokers versus never smokers, in various population subgroups. *Median number of cigarettes per day smoked by current smokers in the baseline data. **Age-sex-standardised rate per 1000 person-years for all subgroups except for subgroups of age and sex, where crude rates are presented. RR (relative risk) adjusted for age, sex, region of residence, alcohol consumption, annual household income and education attainment; adjustment for sex is through stratification in all models except for subgroups of age and income. The dotted line next to the reference line shows the RR of total current smokers compared to total never smokers, see Fig. 1 for details of RR plots and outcome definition

Compared to never smokers, current smokers had a significantly higher risk of AMI in all of the subgroups examined (Fig. 6). The absolute rates of CVD were higher in older versus younger age groups and in men compared to women. Current smoking was more strongly related to AMI in younger compared to older people (Fig. 6), with RRs of 2.93 (2.51–3.41) for age 45–64 and 1.65 (1.25–2.17) for age ≥ 80 years (*p*_interaction_ = 0.0001). Comparing smoking-related RR across grouped CVD outcomes, significantly greater RRs were observed for women versus men for fatal and non-fatal outcomes combined for IHD (*p*_interaction_ = 0.02), including AMI (*p*_interaction_ = 0.007) and non-AMI IHD (*p*_interaction_ = 0.02) but not for stroke, heart failure, peripheral arterial disease, other CVD and any major CVD, nor was any significant sex-related variation apparent for fatal outcomes (Fig. 5, Additional file 2: Figure S8). There was no significant variation in the smoking-related RR according to any of the other factors examined, including according to education, physical activity, diabetes status or alcohol consumption. A greater RR of CVD for current versus never smokers in younger compared to older age groups was observed for chronic ischaemic heart disease and CVD mortality. RRs for CVD mortality in current (versus never) smokers were 3.47 (2.76–4.37) and 2.12 (1.70–2.63) in those aged 45–74 years and ≥ 75 years, respectively; RRs for chronic ischaemic heart disease in current (versus never smokers) were 1.73 (1.60–1.86) and 1.27 (1.08–1.50) in those aged 45–74 years and ≥ 75 years, respectively (data not shown).

The contemporary population-attributable fraction for CVD mortality from smoking was 15% across all age groups, and smoking is estimated to be responsible for around 6400 CVD deaths annually in Australia. The attributable fraction was much higher at younger compared to older ages, being 38.2% in men aged 45–54 decreasing to 9.3% in women aged ≥ 75 years (Table 2); it was 35.5% for the age group 45–64. Overall, 18.3% of hospitalisations for acute coronary syndrome, including AMI, were estimated to be due to smoking, amounting to 11,400 hospitalisations annually. Age-related patterns were similar to mortality with 29.2% of acute coronary syndrome hospitalisations at ages 45–64 years in Australia attributable to smoking. Estimates using 2004–2005 prevalences of smoking, likely to reflect events attributable to smoking during the cohort follow-up period, were similar albeit with somewhat higher SAFs at younger ages (Additional file 2: Table S7).

**Table 2:** Smoking-attributable fractions for CVD mortality and for hospitalisation for acute coronary syndrome in Australia

Age (years)		Observed rate in Australia per 100,000	Prevalence of current smoking in Australia, *p*_cs_	Prevalence of former smoking in Australia, *p*_fs_	Relative risk for current smokers, RR_cs_ (95%CI)	Relative risk for former smokers, RR_fs_ (95%CI)	Smoking-attributable fraction	Smoking-attributable rate/100,000	Smoking-attributable events
CVD mortality									
Males	45–54	63	20.7	37.9	3.47 (2.76–4.37)	1.28 (1.05–1.57)	38.2	24.3	370
	55–64	142	18.3	48.6	3.47 (2.76–4.37)	1.28 (1.05–1.57)	37.1	52.5	684
	65–74	368	11.1	54.1	3.47 (2.76–4.37)	1.28 (1.05–1.57)	30.0	110.1	1013
	≥75	2299	4.0	59.7	2.12 (1.70–2.63)	1.18 (1.06–1.30)	13.0	298.7	1873
Females	45–54	22	17.2	29.4	3.47 (2.76–4.37)	1.28 (1.05–1.57)	33.7	7.5	117
	55–64	52	12.9	33.9	3.47 (2.76–4.37)	1.28 (1.05–1.57)	29.3	15.2	203
	65–74	155	6.9	35.1	3.47 (2.76–4.37)	1.28 (1.05–1.57)	21.3	33.0	311
	≥75	2273	4.5	30.1	2.12 (1.70–2.63)	1.18 (1.06–1.30)	9.3	212.3	1799
Total							14.9		6369
Fatal and non-fatal acute coronary syndrome									
Males	45–54	386	20.7	37.9	2.65 (2.36–2.97)	1.19 (1.08–1.30)	29.2	112.6	1716
	55–64	717	18.3	48.6	2.65 (2.36–2.97)	1.19 (1.08–1.30)	28.2	202.5	2638
	65–74	1127	11.1	54.1	2.65 (2.36–2.97)	1.19 (1.08–1.30)	22.2	250.1	2301
	75–84	1861	4.0	59.7	1.76 (1.42–2.19)	1.19 (1.08–1.31)	12.5	233.3	1101
	≥85	3315	4.0	59.7	1.76 (1.42–2.19)	1.19 (1.08–1.31)	12.5	415.6	645
Females	45–54	125	17.2	29.4	2.65 (2.36–2.97)	1.19 (1.08–1.30)	25.3	31.7	492
	55–64	246	12.9	33.9	2.65 (2.36–2.97)	1.19 (1.08–1.30)	21.7	53.3	710
	65–74	489	6.9	35.1	2.65 (2.36–2.97)	1.19 (1.08–1.30)	15.3	74.6	703
	75–84	1099	4.5	30.1	1.76 (1.42–2.19)	1.19 (1.08–1.31)	8.4	91.8	519
	≥85	2436	4.5	30.1	1.76 (1.42–2.19)	1.19 (1.08–1.31)	8.4	203.6	574
Total							18.3		11,400

Smoking prevalence estimates are from the Australian Health Survey 2014–2015 [19]. Observed rates of CVD mortality are those for all circulatory diseases (rather than any major CVD) in the Australian population [23]. Observed acute coronary syndrome hospitalisations are from national data on hospitalisations for acute myocardial infarction (AMI) or unstable angina in 2013 [24]

RR_cs_ and RR_fs_ used for CVD mortality are the broad age group-specific relative risks for mortality from major CVD among current and former smokers, respectively, relative to never smokers. RR_cs_ and RR_fs_ used for acute coronary syndrome are the broad age group-specific relative risks for fatal and non-fatal AMI among current and former smokers, respectively, relative to never smokers

## Discussion

This study demonstrates the damage that tobacco smoking causes across the entire cardiovascular system. Current tobacco smokers have at least double the risk of developing most significant types of CVD, including AMI, cerebrovascular disease and heart failure, and over five times the risk of developing peripheral arterial disease, compared to people who have never smoked. Mortality from CVD was almost tripled in current versus never smokers. This means that, on average, for current smokers experiencing any of these events, including an AMI, cerebrovascular disease or death from CVD, the balance of probabilities is that it was more likely than not to have been caused by smoking. The relative risks and attributable fractions are greater at younger ages. Around 36% of CVD deaths prior to age 65 and 15% of all CVD deaths in Australia (around 6400 deaths annually)—a country with a relatively low prevalence of current smoking—can be attributed to smoking.

Risks of CVD increased with increasing numbers of cigarettes smoked per day and were greatly diminished among those quitting smoking. Our study showed that what would be generally be regarded as “light smoking” was accompanied by substantial increases in the risk of CVD; smokers of 4–6 cigarettes per day had around double the risk of dying of CVD than people who had never smoked, adding to the emerging evidence on the CVD harms of light smoking [25]. Ex-smokers had substantially lower risks of CVD compared with those continuing to smoke, with those stopping by age 35–44 avoiding around 90% of the excess smoking-attributable risk of CVD conditions such as AMI and stroke.

In common with many other CVD risk factors, relative risks from current smoking are greater at younger ages, with around a tripling in the risk of AMI in current versus never smokers aged 45–64 and a relative risk of 1.65 at age 80 and over. However, because the incidence of CVD is much greater at older ages, the absolute excess CVD incidence attributable to smoking is often greater for older versus younger age groups. For example, the AMI risk difference between current and never smokers is 2.57/1000 person-years at age 45–64 and 7.58/1000 person-years at age 80 years and over. Similarly, while women had lower absolute rates of AMI and non-AMI IHD, the relative risks of these conditions in female current versus never smokers were significantly greater than those seen in their male counterparts. This is in keeping with the published evidence [9]. We did not find any significant variation in the relation of smoking to fatal IHD outcomes, or to CVD outcomes other than IHD, according to sex; we are not aware of other publications examining this. There was no significant variation in the current smoking-AMI relationship according to any of the other population characteristics examined. It is important to bear the absolute risks of CVD in mind when interpreting the findings on relative risks. The absolute number of CVD events attributable to smoking will tend to be greater in groups with higher absolute risks of CVD, including men and those with pre-existing CVD and/or diabetes. Moreover, while the RR in current versus never smokers are greater for peripheral arterial disease than for AMI, the absolute differences in risk between current and never smokers are greater for AMI.

Considering the available published evidence (based on formal searches outlined in the Additional file 1: Brief systematic review of smoking and cardiovascular disease), we conclude that our study presents the most systematic and comprehensive analyses of the relation of smoking to detailed CVD subtypes to date worldwide. It also provides the only contemporary estimates, to our knowledge, from Australia. The study combines detailed prospective data on smoking behaviour and potential confounding factors with virtually complete and independent follow-up of participants for hospitalisation and death, through data linkage. CVD mortality, reported as the underlying cause of death on the death certificate, is therefore captured using this design, as are hospitalisations including CVD as either a primary or secondary diagnosis. For conditions that generally result in hospitalisation and/or death and are well recorded in routine data, such as AMI [26] and cerebrovascular disease [27], combined hospitalisation and mortality data provide sound estimates of incidence. Other conditions, such as angina, chronic IHD, heart failure and dysrhythmias, do not consistently result in hospitalisation or death—and in the case of angina and heart failure, are not generally regarded as legitimate underlying causes of death [28]. In these cases, the rates presented should be considered to reflect hospitalisation with or death from these conditions and not necessarily incidence.

Our study finds, for the first time, a significant increase in the risk of incident paroxysmal tachycardia hospitalisation or death, including for supraventricular tachycardia and ventricular tachycardia, in current versus never smokers; our searches did not locate any comparable studies reporting this,worldwide. These findings are consistent with limited smaller-scale clinical data demonstrating elevated risks in current versus never smokers of ventricular tachyarrhythmias in trial patients with IHD and reduced ejection fraction receiving an implantable cardioverter defibrillator [29], ventricular tachycardia among patients with systolic heart failure [30], and complex arrhythmias in men with existing CVD [31]. Paroxysmal tachycardia (ICD10-AM I47), particularly ventricular tachycardia (I47.2), is potentially life threatening [13, 32]. We also found a relative risk of 1.88 (1.46–2.43) for hospitalisation with or death from cardiomyopathy in current versus never smokers and were unable to locate any directly comparable studies. However, a 1994 publication from the Multiple Risk Factor Intervention Trial found a relative risk of death from idiopathic dilated cardiomyopathy of 1.39 (1.18–1.63) per additional pack of cigarettes smoked per day, using a reference group of men who did not smoke at baseline [33], and a 1993 publication showed statistically compatible findings [34].

Where comparison is possible and bearing in mind the general variation in smoking-related CVD risks according to age, the relative risks observed in this study are broadly consistent with contemporary estimates from countries with similar historical smoking profiles. For example, our finding of a relative risk of IHD mortality of 3.07 (2.48–3.79) in current versus never smokers is consistent with recent US estimates of IHD mortality of 3.0 (2.8–3.2) and 3.5 (2.7–4.6) in women and 2.0 (1.8–2.2) and 3.2 (2.5–4.1) in men [6, 35], and somewhat lower than estimates in the UK women of 4.47 (4.19–4.77) [5]. It is also similar to that seen in the INTERHEART study in 52 countries from 2000 to 2002, which found a relative risk of non-fatal AMI of 2.95 (95%CI 2.77–3.14) in current versus never smokers [36]. Our finding of a relative risk of fatal cerebrovascular disease of 2.26 (1.65–3.10) in current versus never smokers compares to relative risks of 2.1 (1.8–2.3) and 3.2 (2.2–4.7) in women and 1.9 (1.7–2.2) and 1.7 (1.0–2.8) in men from the USA [6, 35] and 3.06 (2.83–3.31) in women from the UK [5]. Our findings of RRs in current versus never smokers for ischaemic stroke (I63) of 2.01 (1.69–2.40) and of intracerebral haemorrhage (I61) of 1.84 (1.33–2.54) are comparable to the figures of 2.17 (2.06–2.28) and 1.77 (1.60–1.95), respectively, from European and North American studies included in a recent meta-analysis [37]. The large > 6-fold smoking-related increases in the risk of abdominal aortic aneurysm and peripheral arterial disease are also consistent with those observed elsewhere for hospitalisation/incidence [7] and mortality [5, 6, 35].

Broad consistency with the published evidence—characterised by overlapping confidence intervals between our estimates and those of the bulk of the published estimates—was observed for our findings in current versus never smokers of 30–40% increases in the risk of atrial fibrillation (Additional file 1: references 14–23) and pulmonary embolism and other venous thrombosis (phlebitis and thrombophlebitis) (Additional file 1: references 3, 24–41). We found a relative risk of 2.23 (1.96–2.53) for heart failure in current versus never smokers; a number of other studies report at least a doubling in risk (Additional file 1: references 42–47), while others report lesser increases in relative risk (Additional file 1: references 48–53). Previous studies consistently show significantly elevated risks of non-rheumatic aortic and mitral valve disorders in current versus never smokers (Additional file 1: references 54–60), with confidence intervals overlapping substantively those of our null results.

Although hypertensive kidney disease and hypertensive heart disease were not significantly associated with smoking in our study, the confidence intervals around these estimates are relatively wide and our point estimates are compatible with findings from a previous study showing significantly elevated mortality from these conditions in current versus never smokers [6].

Composite endpoints, such as “any major CVD” and “IHD”, will necessarily include a different combination of CVD subtypes for fatal and non-fatal outcomes; fatal outcomes will tend to be dominated by mortality from AMI, stroke and heart failure whereas hospitalisation for angina is a common non-fatal outcome (Additional file 2: Table S5). Hence, composite outcomes based on fatal versus non-fatal events are not directly comparable. It follows that studies that investigate smoking-related risks combining multiple CVD subtypes as endpoints (e.g. IHD, combining AMI and angina) are, in effect, summarising potentially heterogeneous RR. Moreover, consistent with previous evidence, our study finds generally higher RR for current versus never smokers for outcomes captured virtually completely by hospitalisation and death data, such as AMI, compared to outcomes which are not, such as angina. The extent to which these differences reflect real differences in the relationship of smoking to disease incidence across subtypes, or under-ascertainment of incidence in those outcomes not well captured, cannot be determined from this study. We therefore emphasise the analyses based on specific subtypes, and the fact that findings relate to hospitalisation and mortality, and, when considering the relationship of smoking to disease incidence, those well captured.

The risks of 29 CVD subtypes were significantly elevated in current compared to never smokers in our study, contributing evidence that few, if any parts of the cardiovascular system remain untouched by smoking, as measured by the occurrence of disease serious enough to cause hospitalisation or death. The strength and consistency of the observed relationship with smoking, the finding of a dose-response relationship and attenuation of risk with quitting are all consistent with the accepted fact that smoking causes CVD. While CVD subtypes tend to be closely related to one another, the underlying pathophysiological mechanisms of these conditions cover the diversity of causes, including atherosclerotic disease, thrombosis, dysrhythmia, hypertension, heart failure and cardiomyopathy.

Our study shows, for the first time to our knowledge, that current smoking confers similarly elevated risks of fatal and non-fatal disease, at least for the outcomes where both incidence and mortality are captured well using data linkage, namely AMI and cerebrovascular disease. Where non-fatal outcomes are unlikely to represent incidence, and smoking-related RRs appear to differ between fatal and non-fatal outcomes, those from the fatal outcomes are likely to be more reliable estimates of the true effect of smoking on disease incidence, as it is not possible in this situation to distinguish between genuinely different RR and differences resulting from issues capturing incidence.

Our findings add to the worldwide evidence that smoking-related risks vary according to CVD subtype, as well as providing independent international confirmation of the magnitude of relative and absolute risks. That such variation is seen within fatal outcomes indicates that it is unlikely to be the result of differential ascertainment or other methodological issues [5, 6].

This study is large and population based, with prospective detailed ascertainment of smoking status from questionnaire items that have been used by the Million Women Study, supporting direct international comparison of results [5]. Since many people now survive a CVD event, a key strength of this study is the inclusion of both fatal and non-fatal events, which provides a more complete and accurate picture of the health effects of smoking. We excluded people with cardiovascular disease and with cancer at baseline, to minimise the impact of smoking cessation due to ill health and to focus on the likely causal effects of smoking. Because of this tendency for smokers—especially older smokers—to quit due to illness, we could not reliably quantify the relationship of smoking cessation after age 55 to the risk of CVD. Overall, 32% of current smokers at baseline who were part of a sample resurveyed 3 years later had quit [16]. This means that the findings presented here are likely to be conservative estimates of the true effect of smoking, apart from the estimates of the relation of CVD to the number of cigarettes smoked each day, where the risks relate more closely to the usual number of cigarettes smoked during follow-up. At the time of the baseline survey, around 12% of individuals aged 45 and over in New South Wales were estimated to be current smokers [38], and following exclusions, around 8% of the 45 and Up Study cohort included in this study were current smokers. Although the 45 and Up Study is, like the vast majority of cohort studies, not designed to be strictly representative of the general population, RR estimates based on internal comparisons within the cohort, such as those presented here, remain valid [38, 39].

The evidence presented here is likely to be of use to the community, policymakers and practitioners in appreciating the magnitude of the harms of smoking across the spectrum of CVD, and the likely benefits of cessation, at the individual and population level; it is especially useful in providing information from a context with low current smoking prevalence and high historical prevalences. CVD is a major cause of morbidity, disability and reduced quality of life, as well as being responsible for large healthcare costs. Our findings strengthen the established evidence on the impact of smoking on CVD mortality and conditions such as ischaemic heart disease, stroke and peripheral vascular disease. They expand the breadth of the known CVD harms of smoking, to include greater evidence on non-fatal disease, smoking relatively few cigarettes per day and on common, high morbidity and mortality conditions such as heart failure, cardiac dysrhythmias and cardiomyopathy. They indicate that quitting smoking and other tobacco control measures are likely to prevent these conditions, reduce healthcare burden and morbidity and improve quality of life. These findings are informative for population-level tobacco control efforts to prevent CVD events and are also likely to be of use clinically, particularly in terms of encouraging quitting in those affected by the CVD conditions identified here as being smoking-related, as well as informing early detection of CVD in current and past smokers.

Because CVD is common and smoking substantially increases the risk of CVD, smoking is responsible for large numbers of cases of CVD, even in countries with extensive tobacco control measures and large declines in smoking prevalence, such as Australia. The prevalence of current adult daily smoking in Australia in 2016 was estimated at 13%, amounting to around 2.7 million current daily smokers; 2% are current smokers who do not smoke on a daily basis and 24% have smoked in the past [40]. Despite the low prevalence, smoking is ranked as the leading or second leading risk factor for burden of disease in Australia [2, 11]; 12% of the CVD burden in 2011 was attributable to smoking [11]. Particularly motivating findings for individuals considering quitting include the observed CVD benefits of quitting at any age, with those quitting by age 45 avoiding almost all of the excess risk, as well as the fact that the balance of probabilities means that a CVD death, myocardial infarction, stroke, hospitalisation with heart failure or aortic aneurysm in a smoker is more likely than not to have been caused by smoking.

## Conclusion

In conclusion, smoking increases the risk of virtually all CVD subtypes, including that of the newly identified risk of paroxysmal tachycardia. Quitting reduces the risk. Smoking accounts for a substantial proportion of premature CVD events in the setting of an established smoking epidemic with low current smoking prevalence.

## Additional files

Additional file 1: Brief systematic review of smoking and cardiovascular disease. (PDF 589 kb)

Additional file 2: **Table S1.** Age-standardised rates of CVD outcomes (fatal and non-fatal combined, and fatal only separately) by sex and smoking status. **Table S2.** Sensitivity analysis for grouped CVD subtypes, in relation to smoking at baseline, excluding the first year of follow-up. A. Combined fatal and non-fatal outcomes. B. Fatal outcomes only. **Table S3.** Sensitivity analysis for combined fatal and non-fatal CVD outcomes, re-classifying past smokers who stopped smoking three or fewer years prior to baseline as current smokers. **Table S4.** Non-fatal outcomes for selected CVD subtypes in relation to smoking status at baseline. **Table S5.** CVD subtypes (level 3 ICD-10-AM codes) ranked according to the frequency of fatal and non-fatal outcomes. **Table S6.** Outcomes for specific CVD subtypes (level 3 ICD-10-AM codes) with at least 50 events, in current and past smokers versus never smokers. A. Combined fatal and non-fatal outcomes. B. Fatal outcomes only. **Table S7.** Smoking-attributable fractions for CVD mortality and hospitalisation for acute coronary syndrome in Australia, using 2004–2005 smoking prevalence estimates. **Table S8.** Selected grouped CVD subtypes in relation to smoking status at baseline by sex. A. Combined fatal and non-fatal outcomes. B. Fatal events only. (PDF 647 kb)

## Data Availability

This study uses data from the 45 and Up Study, which is managed by the Sax Institute in collaboration with major partner Cancer Council NSW, and partners: the National Heart Foundation of Australia (NSW Division); NSW Ministry of Health; NSW Government Family & Community Services–Ageing, Carers and the Disability Council NSW; and the Australian Red Cross Blood Service. Data supporting the findings from this study are available from the Sax Institute, the NSW Department of Health and the Australian Bureau of Statistics, with data linkage conducted by the NSW Centre for Health Record Linkage. Restrictions apply to the availability of these data, which were used under license for the current study, and so are not publicly available. Data are however available from the authors upon reasonable request and with permission of the Sax Institute (www.saxinstitute.org.au) and the NSW Department of Health.

## Abbreviations

AMI: Acute myocardial infarction
CI: Confidence interval
CVD: Cardiovascular disease
HR: Hazard ratio
ICD-10-AM: International Classification of Diseases 10th revision–Australian Modification
IHD: Ischaemic heart disease
PAD: Peripheral arterial disease
RR: Relative risk
SAF: Smoking-attributable fraction
