# Supplementary material for: Tobacco smoking and risk of 36 cardiovascular disease subtypes: fatal and non-fatal outcomes in a large prospective Australian study
Source: BMC Med. 2019 Jul 3;17:128. doi: 10.1186/s12916-019-1351-4 (PMC6607519; doi:10.1186/s12916-019-1351-4)
Supplement: Supplementary file 1 — Brief systematic review of smoking and cardiovascular disease. (PDF 589 kb) [file 12916_2019_1351_MOESM1_ESM.pdf]

TOBACCO SMOKING AND RISK OF 36 CARDIOVASCULAR DISEASE SUBTYPES: FATAL AND NON-FATAL OUTCOMES IN A LARGE PROSPECTIVE AUSTRALIAN STUDY

Banks E, Joshy G, Korda RJ, Stavreski B, Soga K, Egger S, Day C, Clarke NE, Lewington S, Lopez AD

Additional File 1: Brief systematic review of smoking and cardiovascular disease

Additional file 1: Brief systematic review of smoking and cardiovascular disease

Description of methods:

To summarise the worldwide evidence relevant to our findings on the relation of current and past tobacco smoking to cardiovascular disease, we focused initially on large-scale studies and evidence from contexts similar to Australia [Additional file 1: references 1-4] as well as the INTERHEART international study [Additional file 1: reference 5]. For outcomes that were not reported in these large-scale studies, we conducted a systematic search of published evidence to identify any existing larger epidemiological studies examining the relation of smoking to the outcomes of interest. Manual searching of reference lists was undertaken to locate additional publications. Reference lists of all included studies were searched, as well as studies included in relevant recent systematic reviews and meta-analyses [Additional file 1: references 6-11] and a small number of narrative reviews [Additional file 1: references 12-13].

Interpretation:

The list of identified studies is given in this section, below. The results of the studies identified in the systematic review are compared with those of our study, in the discussion section of the main paper. Studies comparing the CVD risks of current smokers with those who have never smoked are emphasised, as these are the comparators in our study. Studies comparing risks in current smokers with people who are not current smokers - grouping past and never smokers together - are used only where there was a lack of evidence using never smokers as the reference group; it should be noted that this is likely to attenuate the relative risk.

Outcomes included in systematic search:

- Atrial fibrillation and flutter
- Cardiomyopathy
- Paroxysmal tachycardia/other arrhythmias
- Heart failure
- Pulmonary embolism
- Thrombophlebitis/phlebitis
- Nonrheumatic mitral valve disorders
- Nonrheumatic aortic valve disorders

Outcomes not included in systematic search, along with reason for non-inclusion:

- Atherosclerosis, aortic aneurysm/dissection, arterial embolism/thrombosis, other peripheral vascular diseases (reported in large-scale studies)
- Cerebrovascular disease outcomes (reported in large-scale studies)
- Ischaemic heart disease outcomes (reported in large-scale studies)
- Vascular disorders of intestine (reported in large-scale studies)
- High blood pressure (reported in large-scale studies)
- Other pulmonary heart diseases (too non-specific)
- Cardiac arrest (too non-specific)
- Complications and ill-defined descriptions of heart disease (too non-specific)

Inclusion criteria:

- Large-scale epidemiological studies in the general population that report at least 100 events for the outcome(s) of interest
- Present a relative risk, hazard ratio, or odds ratio for the relation of **current smoking** to the outcome(s) of interest (fatal and/or non-fatal)

Exclusion criteria:

- Studies in which current and past smokers are grouped together
- Studies of clinical cohorts, including patients with particular diseases, pregnant women, those using specific medications, or those undergoing specific procedures

- Studies in which the clinical end point (outcome) is not clearly defined

Search strategy:

smok* OR tobacco OR cigarette OR cigarettes

AND

“atrial fibrillation” OR “atrial flutter” OR tachycardia OR tachyarrhythmia OR arrhythmia OR dysrhythmia OR cardiomyopathy OR “paroxysmal tachycardia” OR “supraventricular tachycardia” OR “ventricular tachycardia” OR “heart failure” OR “congestive cardiac failure” OR “ventricular failure” OR “ventricular dysfunction” OR “systolic dysfunction” OR “cardiac insufficiency” OR “pulmonary embolism” OR “venous thromboembolism” OR thrombophlebitis OR phlebitis OR “mitral” or “aortic valve” or “aortic stenosis” or “aortic insufficiency”

AND

“risk factor” OR “risk factors” OR predictor OR predictors OR cohort OR prospective OR case-control

Databases:

- PubMed
- Embase

Studies identified from the search:

In total, 78 relevant publications met inclusion criteria for the systematic review. Initial database searches identified 6798 unique papers, and a further 51 potentially relevant papers were identified from reference list searching. 6271 papers were excluded based on title screening, 275 based on abstract screening, and 223 based on full-text screening.

The final list of included studies is shown in the reference list at the end of this Additional file 1 material [Additional file 1: references 14-91]. References cited in the main manuscript are listed alphabetically within the group in which they were cited and groups are listed in order of group citation in the main manuscript. These are followed by uncited references [61-91], listed alphabetically.

Additional file 1 References

1. Carter BD, Abnet CC, Feskanich D, Freedman ND, Hartge P, Lewis CE, et al. Smoking and mortality—beyond established causes. *New Engl J Med* 2015; 372: 631–640.
2. Jha P, Ramasundarahettige C, Landsman V, Rostron B, Thun M, Anderson RN, et al. 21st-century hazards of smoking and benefits of cessation in the United States. *New Engl J Med* 2013; 368: 341–350.
3. Pirie K, Peto R, Reeves GK, Green J, Beral V, Collaborators MWS. The 21st century hazards of smoking and benefits of stopping: a prospective study of one million women in the UK. *Lancet* 2013; 381:133–141.
4. U.S. Department of Health and Human Services. The Health Consequences of Smoking—50 Years of Progress: A Report of the Surgeon General. Atlanta, GA; 2014.
5. Teo KK, Ounpuu S, Hawken S, Pandey M, Valentin V, Hunt D, et al. Tobacco use and risk of myocardial infarction in 52 countries in the INTERHEART study: a case-control study. *Lancet* 2006; 368: 647–658.
6. Aune D, Schlesinger S, Norat T, Riboli E. Tobacco smoking and the risk of atrial fibrillation: a systematic review and meta-analysis of prospective studies. *Eur J Prev Cardiol* 2018; 25: 1437–1451.
7. Cheng Y, Liu Z, Yao F, Zeng W, Zheng D, Dong Y, et al. Current and former smoking and risk for venous thromboembolism: a systematic review and meta-analysis. *PLoS Med* 2013; 10: e1001515.
8. Mi Y, Yan S, Lu Y, Liang Y, Li C. Venous thromboembolism has the same risk factors as atherosclerosis: A PRISMA-compliant systemic review and meta-analysis. *Medicine (Baltimore)* 2016; 95: e4495.
9. Wang Q, Guo Y, Wu C, Yin L, Li W, Shen H, et al. Smoking as a risk factor for the occurrence of atrial fibrillation in men versus women: a meta-analysis of prospective cohort studies. *Heart Lung Circ* 2018; 27: 58–65.
10. Zhang G, Xu X, Su W, Xu Q. Smoking and risk of venous thromboembolism: a systematic review. *Southeast Asian J Trop Med Public Health* 2014; 45:736–745.
11. Zhu W, Yuan P, Shen Y, Wan R, Hong K. Association of smoking with the risk of incident atrial fibrillation: A meta-analysis of prospective studies. *Int J Cardiol* 2016; 218: 259–266.
12. D'Alessandro A, Boeckelmann I, Hammwhoner M, Goette A. Nicotine, cigarette smoking and cardiac arrhythmia: an overview. *Eur J Prev Cardiol* 2012; 19: 297–305.
13. Staerk L, Sherer JA, Ko D, Benjamin EJ, Helm RH. Atrial fibrillation: epidemiology, pathophysiology, and clinical outcomes. *Circ Res* 2017; 120: 1501–1517.
14. Adamsson Eryd S, Ostling G, Rosvall M, Persson M, Smith JG, Melander O, et al. Carotid intima-media thickness is associated with incidence of hospitalized atrial fibrillation. *Atherosclerosis* 2014; 233: 673–678.
15. Chamberlain AM, Agarwal SK, Folsom AR, Duval S, Soliman EZ, Ambrose M, et al. Smoking and incidence of atrial fibrillation: results from the Atherosclerosis Risk in Communities (ARIC) study. *Heart Rhythm* 2011; 8: 1160–1166.
16. Diouf I, Magliano DJ, Carrington MJ, Stewart S, Shaw JE. Prevalence, incidence, risk factors and treatment of atrial fibrillation in Australia: The Australian Diabetes, Obesity and Lifestyle (AusDiab) longitudinal, population cohort study. *Int J Cardiol* 2016; 205: 127–132.
17. Frost L, Hune LJ, Vestergaard P. Overweight and obesity as risk factors for atrial fibrillation or flutter: the Danish Diet, Cancer, and Health Study. *Am J Med* 2005; 118: 489–495.
18. Heeringa J, Kors JA, Hofman A, van Rooij FJA, Witteman JCM. Cigarette smoking and risk of atrial fibrillation: the Rotterdam Study. *Am Heart J* 2008; 156: 1163–1169.
19. Imtiaz Ahmad M, Mosley CD, O'Neal WT, Judd SE, McClure LA, Howard VJ, et al. Smoking and risk of atrial fibrillation in the REasons for Geographic And Racial Differences in Stroke (REGARDS) study. *J Cardiol* 2018; 71: 113–117.
20. Knuiman M, Briffa T, Divitini M, Chew D, Eikelboom J, McQuillan B, et al. A cohort study examination of established and emerging risk factors for atrial fibrillation: the Busselton Health Study. *Eur J Epidemiol* 2014; 29: 181–190.
21. Kokubo Y, Watanabe M, Higashiyama A, Nakao YM, Kusano K, Miyamoto Y. Development of a basic risk score for incident atrial fibrillation in a Japanese general population: the Suita study. *Circ J* 2017; 81: 1580–1588.
22. Pfister R, Bragelmann J, Michels G, Wareham NJ, Luben R, Khaw KT. Performance of the CHARGE-AF risk model for incident atrial fibrillation in the EPIC Norfolk cohort. *Eur J Prev Cardiol* 2015; 22: 932–939.
23. Zuo H, Nygard O, Vollset SE, Ueland PM, Ulvik A, Midttun O, et al. Smoking, plasma cotinine and risk of atrial fibrillation: the Hordaland Health Study. *J Intern Med* 2018; 283: 73–82.
24. Blondon M, Wiggins KL, McKnight B, Psaty BM, Rice KM, Heckbert SR, et al. The association of smoking with venous thrombosis in women. A population-based, case-control study. *Thromb Haemost* 2013; 109: 891–896.

25. Crous-Bou M, De Vivo I, Camargo CA Jr, Varraso R, Grodstein F, Jensen MK, et al. Interactions of established risk factors and a GWAS-based genetic risk score on the risk of venous thromboembolism. *Thromb Haemost* 2016; 116: 705–713.
26. Enga KF, Braekkan SK, Hansen-Krone IJ, Le Cessie S, Rosendaal FR, Hansen JB. Cigarette smoking and the risk of venous thromboembolism: the Tromsø study. *J Thromb Haemost* 2012; 10: 2068–2074.
27. Glynn RJ, Rosner B. Comparison of risk factors for the competing risks of coronary heart disease, stroke, and venous thromboembolism. *Am J Epidemiol* 2005; 162: 975–982.
28. Goldhaber SZ, Grodstein F, Stampfer MJ, Manson JE, Colditz GA, Speizer FE, et al. A prospective study of risk factors for pulmonary embolism in women. *JAMA* 1997; 277: 642–645.
29. Heit JA, Silverstein MD, Mohr DN, Petterson TM, O'Fallon WM, Melton LJ. Risk factors for deep vein thrombosis and pulmonary embolism: a population-based case-control study. *Arch Intern Med* 2000; 160: 809–815.
30. Hippisley-Cox J, Coupland C. Development and validation of risk prediction algorithm (QThrombosis) to estimate future risk of venous thromboembolism: prospective cohort study. *BMJ* 2011; 343: d4656.
31. Holst AG, Jensen G, Prescott E. Risk factors for venous thromboembolism: results from the Copenhagen City Heart Study. *Circulation* 2010; 121: 1896–1903.
32. Klatsky AL, Armstrong MA, Poggi J. Risk of pulmonary embolism and/or deep venous thrombosis in Asian-Americans. *Am J Cardiol* 2000; 85: 1334–1337.
33. Lutsey PL, Virnig BA, Durham SB, Steffen LM, Hirsch AT, Jacobs DR Jr, et al. Correlates and consequences of venous thromboembolism: The Iowa Women's Health Study. *Am J Public Health* 2010; 100: 1506–1513.
34. Mahmoodi BK, Cushman M, Anne Naess I, Allison MA, Bos WJ, Braekkan SK, et al. Association of traditional cardiovascular risk factors with venous thromboembolism: an individual participant data meta-analysis of prospective studies. *Circulation* 2017; 135: 7–16.
35. Pomp ER, Rosendaal FR, Doggen CJ. Smoking increases the risk of venous thrombosis and acts synergistically with oral contraceptive use. *Am J Hematol* 2008; 83: 97–102.
36. Rosengren A, Freden M, Hansson PO, Wilhelmsen L, Wedel H, Eriksson H. Psychosocial factors and venous thromboembolism: a long-term follow-up study of Swedish men. *J Thromb Haemost* 2008; 6: 558–564.
37. Severinsen MT, Kristensen SR, Johnsen SP, Dethlefsen C, Tjonneland A, Overvad K. Smoking and venous thromboembolism: a Danish follow-up study. *J Thromb Haemost* 2009; 7: 1297–1303.
38. Sugimura K, Sakuma M, Shirato K. Potential risk factors and incidence of pulmonary thromboembolism in Japan: results from an overview of mailed questionnaires and matched case-control study. *Circ J* 2006; 70: 542–547.
39. Sweetland S, Parkin L, Balkwill A, Green J, Reeves G, Beral V, et al. Smoking, surgery, and venous thromboembolism risk in women: United Kingdom cohort study. *Circulation* 2013; 127: 1276–1282.
40. Tsai AW, Cushman M, Rosamond WD, Heckbert SR, Polak JF, Folsom AR. Cardiovascular risk factors and venous thromboembolism incidence: the longitudinal investigation of thromboembolism etiology. *Arch Intern Med* 2002; 162: 1182–1189.
41. Wattanakit K, Lutsey PL, Bell EJ, Gornik H, Cushman M, Heckbert SR, et al. Association between cardiovascular disease risk factors and occurrence of venous thromboembolism: a time-dependent analysis. *Thromb Haemost* 2012; 108: 508–515.
42. Butler J, Kalogeropoulos A, Georgiopoulou V, Belue R, Rodondi N, Garcia M, et al. Incident heart failure prediction in the elderly: the health ABC heart failure score. *Circ Heart Fail* 2008; 1: 125–133.
43. Chahal H, Bluemke DA, Wu CO, McClelland R, Liu K, Shea SJ, et al. Heart failure risk prediction in the Multi-Ethnic Study of Atherosclerosis. *Heart* 2015; 101: 58–64.
44. Johansson S, Wallander MA, Ruigomez A, Garcia Rodriguez LA. Incidence of newly diagnosed heart failure in UK general practice. *Eur J Heart Fail* 2001; 3: 225–231.
45. Kamimura D, Cain LR, Mentz RJ, White WB, Blaha MJ, DeFilippis AP, et al. Cigarette smoking and incident heart failure: insights from the Jackson Heart Study. *Circulation* 2018; 137: 2572–2582.
46. Sahle BW, Owen AJ, Krum H, Reid CM, Second Australian National Blood Pressure Study Management Committee. Incidence of heart failure in 6083 elderly hypertensive patients: the Second Australian National Blood Pressure Study (ANBP2). *Eur J Heart Fail* 2016; 18: 38–45.
47. Wang Y, Tuomilehto J, Jousilahti P, Antikainen R, Mahonen M, Katzmarzyk PT, et al. Lifestyle factors in relation to heart failure among Finnish men and women. *Circ Heart Fail* 2011; 4: 607–612.
48. Ahmed AA, Patel K, Nyaku MA, Kheirbek RE, Bittner V, Fonarow GC, Filippatos GS, et al. Risk of heart failure and death after prolonged smoking cessation: role of amount and duration of prior smoking. *Circ Heart Fail* 2015; 8: 694–701.

49. Del Gobbo LC, Kalantarian S, Imamura F, Lemaitre R, Siscovick DS, Psaty BM, et al. Contribution of major lifestyle risk factors for incident heart failure in older adults: the Cardiovascular Health Study. *JACC Heart Fail* 2015; 3: 520–528.
50. He J, Ogden LG, Bazzano LA, Vupputuri S, Loria C, Whelton PK. Risk factors for congestive heart failure in US men and women: NHANES I epidemiologic follow-up study. *Arch Intern Med* 2001; 161: 996–1002.
51. Pujades-Rodriguez M, George J, Shah AD, Rapsomaniki E, Denaxas S, West R, et al. Heterogeneous associations between smoking and a wide range of initial presentations of cardiovascular disease in 1937360 people in England: lifetime risks and implications for risk prediction. *Int J Epidemiol* 2015; 44: 129–141.
52. Tran B, Falster MO, Douglas K, Blyth F, Jorm LR. Smoking and potentially preventable hospitalisation: the benefit of smoking cessation in older ages. *Drug Alcohol Depend* 2015; 150: 85–91.
53. White JR, Chang CH, So-Armah KA, Stewart JC, Gupta SK, Butt AA, et al. Depression and human immunodeficiency virus infection are risk factors for incident heart failure among veterans veterans aging cohort study. *Circulation* 2015; 132: 1630–1638.
54. Elmariah S, Budoff MJ, Delaney JAC, Hamirani Y, Eng J, Fuster V, et al. Risk factors associated with the incidence and progression of mitral annulus calcification: the multi-ethnic study of atherosclerosis. *Am Heart J* 2013; 166: 904–912.
55. Eveborn GW, Schirmer H, Lunde P, Heggelund G, Hansen J-B, Rasmussen K. Assessment of risk factors for developing incident aortic stenosis: the Tromso Study. *Eur J Epidemiol* 2014; 29: 567–575.
56. Hoagland PM, Cook EF, Flatley M, Walker C, Goldman L. Case-control analysis of risk factors for presence of aortic stenosis in adults (age 50 years or older). *Am J Cardiol* 1985; 55: 744–747.
57. Larsson SC, Wolk A, Back M. Alcohol consumption, cigarette smoking and incidence of aortic valve stenosis. *J Intern Med* 2017; 282: 332–339.
58. Martinsson A, Ostling G, Persson M, Sundquist K, Andersson C, Melander O, et al. Carotid plaque, intima-media thickness, and incident aortic stenosis: a prospective cohort study. *Arterioscler Thromb Vasc Biol* 2014; 34: 2343–2348.
59. Stewart BF, Siscovick D, Lind BK, Gardin JM, Gottdiener JS, Smith VE, et al. Clinical factors associated with calcific aortic valve disease. Cardiovascular Health Study. *J Am Coll Cardiol* 1997; 29: 630–634.
60. Thanassoulis G, Massaro JM, Cury R, Manders E, Benjamin EJ, Vasan RS, et al. Associations of long-term and early adult atherosclerosis risk factors with aortic and mitral valve calcium. *J Am Coll Cardiol* 2010; 55: 2491–2498.
61. Abramson J, Berger A, Krumholz HM, Vaccarino V. Depression and risk of heart failure among older persons with isolated systolic hypertension. *Arch Intern Med* 2001; 161: 1725–1730.
62. Abramson JL, Williams SA, Krumholz HM, Vaccarino V. Moderate alcohol consumption and risk of heart failure among older persons. *JAMA* 2001; 285: 1971–1977.
63. Albert CM, Chae CU, Grodstein F, Rose LM, Rexrode KM, Ruskin JN, et al. Prospective study of sudden cardiac death among women in the United States. *Circulation* 2003; 107: 2096–2101.
64. Alonso A, Krijthe BP, Aspelund T, Stepas KA, Pencina MJ, Moser CB, et al. Simple risk model predicts incidence of atrial fibrillation in a racially and geographically diverse population: the CHARGE-AF consortium. *J Am Heart Assoc* 2013; 2: e000102.
65. Austin TR, Wiggins KL, Blackshear C, Yang Y, Benjamin EJ, Curtis LH, et al. Atrial fibrillation in an African-American cohort: the Jackson Heart Study. *Clin Cardiol* 2018; in press. doi: 10.1002/clc.23020.
66. Benjamin EJ, Levy D, Vaziri SM, D'Agostino RB, Belanger AJ, Wolf PA. Independent risk factors for atrial fibrillation in a population-based cohort. The Framingham Heart Study. *JAMA* 1994; 271: 840–844.
67. Brouwers FP, de Boer RA, van der Harst P, Voors AA, Gansevoort RT, Bakker SJ, et al. Incidence and epidemiology of new onset heart failure with preserved vs. reduced ejection fraction in a community-based cohort: 11-year follow-up of PREVEND. *Eur Heart J* 2013; 34: 1424–1431.
68. Coughlin SS, Labenberg JR, Tefft MC. Black-white differences in idiopathic dilated cardiomyopathy: the Washington DC dilated Cardiomyopathy Study. *Epidemiology* 1993; 4: 165–172.
69. Coughlin SS, Neaton JD, Sengupta A, Kuller LH. Predictors of mortality from idiopathic dilated cardiomyopathy in 356,222 men screened for the Multiple Risk Factor Intervention Trial. *Am J Epidemiol* 1994; 139: 166–172.
70. Eaton CB, Pettinger M, Rossouw J, Martin LW, Foraker R, Quddus A, et al. Risk factors for incident hospitalized heart failure with preserved versus reduced ejection fraction in a multiracial cohort of postmenopausal women. *Circ Heart Fail* 2016; 9: e002883.
71. Eriksson H, Svardsudd K, Larsson B, Ohlson LO, Tibblin G, Welin L, et al. Risk factors for heart failure in the general population: the study of men born in 1913. *Eur Heart J* 1989; 10: 647–656.

72. Gronich N, Lavi I, Rennert G. Higher risk of venous thrombosis associated with drospirenone-containing oral contraceptives: a population-based cohort study. *CMAJ* 2011; 183: E1319-25.
73. Haywood LJ, Ford CE, Crow RS, Davis BR, Massie BM, Einhorn PT, et al. Atrial fibrillation at baseline and during follow-up in ALLHAT (Antihypertensive and Lipid-Lowering Treatment to Prevent Heart Attack Trial). *J Am Coll Cardiol* 2009; 54: 2023–2031.
74. Ho JE, Enserro D, Brouwers FP, Kizer JR, Shah SJ, Psaty BM, et al. Predicting heart failure with preserved and reduced ejection fraction: the international collaboration on heart failure subtypes. *Circ Heart Fail* 2016; 9: e003116.
75. Ho JE, Lyass A, Lee DS, Vasan RS, Kannel WB, Larson MG, et al. Predictors of new-onset heart failure: differences in preserved versus reduced ejection fraction. *Circ Heart Fail* 2013; 6: 279–286.
76. Ingelsson E, Arnlov J, Lind L, Sundstrom J. Metabolic syndrome and risk for heart failure in middle-aged men. *Heart* 2006; 92: 1409–1413.
77. Ingelsson E, Sundstrom J, Arnlov J, Zethelius B, Lind L. Insulin resistance and risk of congestive heart failure. *JAMA* 2005; 294: 334–341.
78. Jacobs L, Efremov L, Ferreira JP, Thijs L, Yang W-Y, Zhang Z-Y, et al. Risk for incident heart failure: a subject-level meta-analysis from the Heart "OMics" in AGEing (HOMAGE) study. *J Am Heart Assoc* 2017; 6: e005231.
79. Krahn AD, Manfreda J, Tate RB, Mathewson FA, Cuddy TE. The natural history of atrial fibrillation: incidence, risk factors, and prognosis in the Manitoba Follow-Up Study. *Am J Med* 1995; 98: 476–484.
80. Li Z, Yadav U, Mahemuti A, Tang B-P, Upur H. Association of MTHFR genetic polymorphisms with venous thromboembolism in Uyghur population in Xinjiang, China. *Int J Clin Exp Med* 2015; 8: 17703–17711.
81. Magnussen C, Niiranen TJ, Ojeda FM, Gianfagna F, Blankenberg S, Njolstad I, et al. Sex differences and similarities in atrial fibrillation epidemiology, risk factors, and mortality in community cohorts: results from the BiomarCaRE consortium (Biomarker for Cardiovascular Risk Assessment in Europe). *Circulation* 2017; 136: 1588-1597.
82. Mahmoodi BK, Gansevoort RT, Veeger NJ, Matthews AG, Navis G, Hillege HL, et al. Microalbuminuria and risk of venous thromboembolism. *JAMA* 2009; 301: 1790–1797.
83. Nightingale AL, Lawrenson RA, Simpson EL, Williams TJ, MacRae KD, Farmer RD. The effects of age, body mass index, smoking and general health on the risk of venous thromboembolism in users of combined oral contraceptives. *Eur J Contracept Reprod Health Care* 2000; 5: 265–274.
84. Poulter N, Chang C, Farley T, Meirik O, Marmot M. Venous thromboembolic disease and combined oral contraceptives: results of international multicentre case-control study. *Lancet* 1995; 346: 1575–1582.
85. Quist-Paulsen P, Næss IA, Cannegieter SC, Romundstad PR, Christiansen SC, Rosendaal FR, et al. Arterial cardiovascular risk factors and venous thrombosis: results from a population based prospective study (the HUNT 2). *Haematologica* 2009; 95: 119–125.
86. Rahman F, Wang N, Yin X, Ellinor PT, Lubitz SA, LeLorier PA, et al. Atrial flutter: clinical risk factors and adverse outcomes in the Framingham Heart Study. *Heart Rhythm* 2016; 13: 233–240.
87. Roberts CB, Couper DJ, Chang PP, James SA, Rosamond WC, Heiss G. Influence of life-course socioeconomic position on incident heart failure in blacks and whites. *Am J Epidemiol* 2010; 172: 717–727.
88. Schnabel RB, Sullivan LM, Levy D, Pencina MJ, Massaro JM, D'Agostino RB Sr. Development of a risk score for atrial fibrillation (Framingham Heart Study): a community-based cohort study. *Lancet* 2009; 373: 739–745.
89. Schnabel RB, Yin X, Gona P, Larson MG, Beiser AS, McManus DD, et al. 50 year trends in atrial fibrillation prevalence, incidence, risk factors, and mortality in the Framingham Heart Study: a cohort study. *Lancet* 2015; 386: 154–162.
90. Smith JG, Platonov PG, Hedblad B, Engström G, Melander O. Atrial fibrillation in the Malmö Diet and Cancer study: a study of occurrence, risk factors and diagnostic validity. *Eur J Epidemiol* 2010; 25: 95–102.
91. Wilhelmsen L, Rosengren A, Eriksson H, Lappas G. Heart failure in the general population of men-morbidity, risk factors and prognosis. *J Intern Med* 2001; 249: 25
