# Supplementary material for: Tobacco smoking and risk of 36 cardiovascular disease subtypes: fatal and non-fatal outcomes in a large prospective Australian study
Source: BMC Med. 2019 Jul 3;17:128. doi: 10.1186/s12916-019-1351-4 (PMC6607519; doi:10.1186/s12916-019-1351-4)
Supplement: Supplementary file 2 — Table S1. Age-standardised rates of CVD outcomes (fatal and non-fatal combined, and fatal only separately) by sex and smoking status. Table S2. Sensitivity analysis for grouped CVD subtypes, in relation to smoking at baseline, excluding the first year of follow-up. A. Combined fatal and non-fatal outcomes. B. Fatal outcomes only. Table S3. Sensitivity analysis for combined fatal and non-fatal CVD outcomes, re-classifying past smokers who stopped smoking three or fewer years prior to baseline as current smokers. Table S4. Non-fatal outcomes for selected CVD subtypes in relation to smoking status at baseline. Table S5. CVD subtypes (level 3 ICD-10-AM codes) ranked according to the frequency of fatal and non-fatal outcomes. Table S6. Outcomes for specific CVD subtypes (level 3 ICD-10-AM codes) with at least 50 events, in current and past smokers versus never smokers. A. Combined fatal and non-fatal outcomes. B. Fatal outcomes only. Table S7. Smoking-attributable fractions for CVD mortality and hospitalisation for acute coronary syndrome in Australia, using 2004–2005 smoking prevalence estimates. Table S8. Selected grouped CVD subtypes in relation to smoking status at baseline by sex. A. Combined fatal and non-fatal outcomes. B. Fatal events only. (PDF 647 kb) [file 12916_2019_1351_MOESM2_ESM.pdf]

TOBACCO SMOKING AND RISK OF 36 CARDIOVASCULAR DISEASE SUBTYPES: FATAL AND NON-FATAL OUTCOMES IN A LARGE PROSPECTIVE AUSTRALIAN STUDY

Banks E, Joshy G, Korda RJ, Stavreski B, Soga K, Egger S, Day C, Clarke NE, Lewington S, Lopez AD

Additional File 2: Supplementary tables and figures

Index

Table S1. Age standardised rates of CVD outcomes (fatal and non-fatal combined, and mortality separately) by sex and smoking status 3

Table S2: Outcomes for grouped CVD subtypes, in relation to smoking at baseline, excluding the first year of follow up 4

A. Combined fatal and non-fatal outcomes 4

B. Fatal outcomes only 5

Table S3. Sensitivity analysis for combined fatal and non-fatal CVD outcomes, re-classifying past smokers who stopped smoking three or fewer years prior to baseline as current smokers. 6

Table S4: Non-fatal outcomes for selected CVD subtypes in relation to smoking status at baseline 7

Table S5. CVD subtypes (level 3 ICD-10 AM codes) ranked according to frequency of fatal and non-fatal outcomes 8

Table S6: Outcomes for specific CVD subtypes (level 3 ICD-10-AM codes) with at least 50 events, in current and past smokers versus never smokers 9

A. Combined fatal and non-fatal outcomes 9

B. Fatal outcomes only 10

Table S7. Smoking attributable fractions for CVD mortality and hospitalisation for acute coronary syndrome in Australia, using 2004-5 smoking prevalence estimates 11

Table S8. Selected grouped CVD subtypes in relation to smoking status at baseline by sex 12

A.Combined fatal and non-fatal events 12

B.Fatal events only 13

Table S1. Age standardised rates of CVD outcomes (fatal and non-fatal combined, and fatal only separately) by sex and smoking status

	Fatal and non-fatal outcomes										Fatal outcomes only									
	Men					Women					Men					Women				
	Events	PY*	Std rate**	95% CI		Events	PY*	Std rate**	95% CI		Events	PY*	Std rate**	95% CI		Events	PY*	Std rate**	95% CI	
**Ischaemic heart disease (IHD)**																				
Never	3284	311.0	10.28	9.91	10.68	2908	511.7	5.25	5.05	5.46	268	322.3	0.90	0.80	1.03	306	521.0	0.54	0.48	0.62
Past	3313	243.0	11.55	11.10	12.04	1208	224.8	5.87	5.51	6.25	314	254.0	1.06	0.95	1.22	100	228.7	0.62	0.50	0.77
Current	691	51.1	14.83	13.45	16.37	374	56.9	9.02	7.84	10.38	76	53.5	2.34	1.73	3.15	37	58.1	1.14	0.71	1.78
**Acute myocardial infarction (AMI)**																				
Never	1231	318.5	3.87	3.64	4.12	1087	518.2	1.96	1.84	2.10	132	322.3	0.46	0.38	0.56	174	521.0	0.31	0.27	0.37
Past	1244	250.3	4.30	4.03	4.61	443	227.5	2.29	2.07	2.54	145	254.0	0.49	0.42	0.62	56	228.7	0.34	0.26	0.46
Current	365	52.3	7.98	6.94	9.18	178	57.6	4.61	3.75	5.65	34	53.5	1.01	0.62	1.61	17	58.1	0.52	0.25	1.00
**IHD, excluding AMI**																				
Never	2942	311.7	9.02	8.67	9.39	2428	512.7	4.33	4.15	4.52	136	322.3	0.44	0.37	0.54	132	521.0	0.23	0.19	0.28
Past	2956	243.7	10.18	9.75	10.63	1034	225.1	4.89	4.57	5.24	169	254.0	0.57	0.48	0.69	44	228.7	0.28	0.20	0.39
Current	606	51.3	12.45	11.25	13.82	312	57.0	6.91	5.94	8.05	42	53.5	1.33	0.88	1.98	20	58.1	0.62	0.32	1.14
**Cerebrovascular disease**																				
Never	951	319.9	3.17	2.96	3.39	1182	518.1	2.17	2.04	2.31	161	322.3	0.58	0.50	0.69	252	521.0	0.46	0.40	0.52
Past	1048	251.3	3.53	3.31	3.78	439	227.6	2.31	2.08	2.56	172	254.0	0.60	0.51	0.73	67	228.7	0.42	0.32	0.55
Current	231	52.8	6.15	5.16	7.31	140	57.7	4.00	3.18	5.01	28	53.5	1.16	0.70	1.84	17	58.1	0.74	0.38	1.32
**Heart failure**																				
Never	781	320.7	2.71	2.51	2.92	1201	518.5	2.21	2.09	2.35	30	322.3	0.11	0.07	0.17	73	521.0	0.13	0.10	0.17
Past	1089	251.6	3.74	3.52	4.00	498	227.6	2.85	2.60	3.14	49	254.0	0.17	0.12	0.27	19	228.7	0.11	0.07	0.19
Current	186	53.1	5.34	4.41	6.47	119	57.8	4.67	3.69	5.85	<5	53.5	0.12	0.02	0.47	5	58.1	0.31	0.09	0.77
**Peripheral arterial disease**																				
Never	432	321.0	1.39	1.25	1.54	472	519.6	0.85	0.77	0.94	25	322.3	0.09	0.06	0.15	25	521.0	0.04	0.03	0.08
Past	730	251.7	2.50	2.29	2.74	285	227.8	1.47	1.29	1.68	45	254.0	0.15	0.11	0.24	18	228.7	0.11	0.07	0.20
Current	254	52.7	5.88	5.01	6.93	138	57.7	4.02	3.21	5.03	15	53.5	0.40	0.18	0.83	7	58.1	0.33	0.11	0.78
**Other major CVD**																				
Never	3448	311.6	11.19	10.79	11.61	4630	506.4	8.59	8.33	8.87	107	322.3	0.36	0.30	0.45	142	521.0	0.25	0.21	0.31
Past	3476	243.4	12.27	11.82	12.75	1878	222.8	9.47	9.00	9.96	108	254.0	0.37	0.30	0.48	41	228.7	0.24	0.17	0.34
Current	562	52.0	14.16	12.71	15.79	406	57.0	10.78	9.42	12.34	17	53.5	0.63	0.34	1.14	13	58.1	0.61	0.29	1.16
**ANY MAJOR CVD**																				
**Never**	6790	299.8	23.57	22.96	24.19	8038	495.2	15.72	15.36	16.09	591	322.3	2.04	1.88	2.23	798	521.0	1.42	1.33	1.53
**Past**	7027	231.0	27.10	26.39	27.84	3325	218.0	17.79	17.12	18.48	688	254.0	2.34	2.17	2.55	245	228.7	1.51	1.32	1.72
**Current**	1437	48.9	37.47	34.92	40.23	894	55.3	24.57	22.39	26.97	139	53.5	4.66	3.75	5.76	79	58.1	3.12	2.33	4.12

*1,000 person-years. **Age standardised rate per 1000 person-years. Ischaemic heart disease (IHD) (I20-I25); Acute myocardial infarction (I21); IHD, excluding AMI (I20, I22-I25); Cerebrovascular disease (I61-I67 and I69); Heart failure (I50); Peripheral arterial disease (I70-I74); Other major CVD (any major CVD except ischaemic heart disease, cerebrovascular disease, heart failure, and peripheral arterial disease); Any major CVD as defined in: Joshy G et al. Categorising major cardiovascular disease hospitalisations from routinely collected data. *Public Health Research & Practice*. 2015; 25(3): e2531532.

Table S2: Sensitivity analysis for grouped CVD subtypes, in relation to smoking at baseline, excluding the first year of follow up

A. Combined fatal and non-fatal outcomes

	Events	Std rate*	RR (95% CI)
**Ischaemic heart disease (IHD)**			
Never smoker	5449	7.47	1.00
Past smoker	3983	8.47	1.17 (1.12-1.22)
Current smoker	928	11.91	1.66 (1.54-1.78)
**Acute myocardial infarction (AMI)**			
Never	2039	2.74	1.00
Past	1493	3.23	1.23 (1.15-1.32)
Current	487	6.28	2.54 (2.29-2.81)
**IHD, excluding AMI**			
Never	4725	6.45	1.00
Past	3517	7.33	1.17 (1.12-1.22)
Current	796	9.63	1.57 (1.45-1.69)
**Cerebrovascular disease**			
Never smoker	1896	2.56	1.00
Past smoker	1352	2.86	1.15 (1.07-1.24)
Current smoker	336	5.11	2.23 (1.98-2.52)
**Heart failure**			
Never smoker	1830	2.45	1.00
Past smoker	1453	3.24	1.44 (1.34-1.56)
Current smoker	293	5.32	2.30 (2.02-2.62)
**Peripheral arterial disease**			
Never smoker	804	1.06	1.00
Past smoker	906	1.92	1.79 (1.62-1.97)
Current smoker	364	5.09	5.32 (4.67-6.07)
**Other major CVD**			
Never smoker	7267	9.85	1.00
Past smoker	4804	10.94	1.09 (1.05-1.14)
Current smoker	873	12.57	1.25 (1.16-1.34)
**ANY MAJOR CVD**			
**Never smoker**	13032	19.11	1.00
**Past smoker**	9082	22.04	1.17 (1.13-1.20)
**Current smoker**	2055	30.90	1.65 (1.57-1.73)

*Age-sex standardised rate per 1000 person-years.
RR (relative risk) adjusted for age, sex, region of residence, alcohol consumption, annual household income, and education attainment; adjustment for sex is through stratification for outcomes IHD, AMI, non-AMI, IHD, heart failure (stratified for income as well) and major CVD. For definitions of outcomes see Additional file 2: Table S1.

B. Fatal outcomes only

	Events	Std rate*	RR (95% CI)
**Ischaemic heart disease (IHD)**			
Never smoker	535	0.71	1.00
Past smoker	381	0.82	1.25 (1.08-1.44)
Current smoker	99	1.73	2.93 (2.34-3.67)
**Acute myocardial infarction (AMI)**			
Never	283	0.38	1.00
Past	177	0.39	1.11 (0.91-1.36)
Current	45	0.74	2.72 (1.96-3.79)
**IHD, excluding AMI**			
Never	252	0.33	1.00
Past	204	0.42	1.34 (1.10-1.63)
Current	54	0.99	3.15 (2.31-4.29)
**Cerebrovascular disease**			
Never smoker	396	0.52	1.00
Past smoker	233	0.53	1.04 (0.87-1.23)
Current smoker	44	0.95	2.31 (1.68-3.18)
**ANY MAJOR CVD**			
**Never smoker**	1311	1.72	1.00
**Past smoker**	880	1.91	1.20 (1.10-1.32)
**Current smoker**	199	4.05	2.68 (2.29-3.13)

*Age-sex standardised rate per 1000 person-years.
RR (relative risk) adjusted for age, sex, region of residence, alcohol consumption, annual household income, and education attainment; adjustment for sex is through stratification for outcomes IHD and major CVD. For definitions of outcomes see Additional file 2: Table S1.

Table S3. Sensitivity analysis for combined fatal and non-fatal CVD outcomes, re-classifying past smokers who stopped smoking three or fewer years prior to baseline as current smokers.

	Events	Std rate*	RR (95% CI)
**Ischaemic heart disease (IHD)**			
Never smoker	6192	7.57	1.00
Past smoker	4229	8.39	1.13 (1.09-1.18)
Current smoker	1357	11.52	1.65 (1.55-1.75)
**Acute myocardial infarction (AMI)**			
Never	2318	2.84	1.00
Past	1575	3.16	1.18 (1.10-1.26)
Current	655	5.78	2.33 (2.13-2.55)
**IHD, excluding AMI**			
Never	5370	6.51	1.00
Past	3728	7.26	1.13 (1.09-1.18)
Current	1180	9.40	1.58 (1.48-1.68)
**Cerebrovascular disease**			
Never smoker	2133	2.58	1.00
Past smoker	1395	2.77	1.09 (1.01-1.17)
Current smoker	463	4.79	2.15 (1.93-2.39)
**Heart failure**			
Never smoker	1982	2.41	1.00
Past smoker	1492	3.16	1.42 (1.32-1.52)
Current smoker	400	5.17	2.37 (2.12-2.66)
**Peripheral arterial disease**			
Never smoker	904	1.08	1.00
Past smoker	947	1.87	1.72 (1.57-1.90)
Current smoker	460	4.63	4.71 (4.18-5.30)
**Other major CVD**			
Never smoker	8078	9.76	1.00
Past smoker	5075	10.67	1.09 (1.05-1.13)
Current smoker	1247	12.21	1.26 (1.19-1.34)
**ANY MAJOR CVD**			
**Never smoker**	14828	19.26	1.00
**Past smoker**	9718	21.72	1.14 (1.11-1.17)
**Current smoker**	2965	30.10	1.63 (1.57-1.70)

*Age-sex standardised rate per 1000 person-years.
RR (relative risk) adjusted for age, sex, region of residence, alcohol consumption, annual household income, and education attainment; adjustment for sex is through stratification for outcomes IHD, AMI, non-AMI, IHD, heart failure (stratified for income as well) and major CVD. For definitions of outcomes see Additional file 2: Table S1.

Table S4: Non-fatal outcomes for selected CVD subtypes in relation to smoking status at baseline

	Events	Std rate*	RR (95% CI)
**Ischaemic heart disease (IHD)**			
Never smoker	5886	7.17	1.00
Past smoker	4281	8.03	1.15 (1.10-1.19)
Current smoker	990	10.74	1.56 (1.45-1.67)
**Acute myocardial infarction (AMI)**			
Never	2144	2.62	1.00
Past	1564	2.96	1.20 (1.12-1.29)
Current	508	5.74	2.41 (2.17-2.66)
**IHD, excluding AMI**			
Never	5190	6.28	1.00
Past	3842	7.08	1.15 (1.10-1.20)
Current	874	8.87	1.53 (1.42-1.65)
**Cerebrovascular disease**			
Never smoker	1988	2.40	1.00
Past smoker	1402	2.63	1.12 (1.05-1.21)
Current smoker	359	4.66	2.16 (1.93-2.43)
**Heart failure**			
Never smoker	1941	2.37	1.00
Past smoker	1562	3.19	1.47 (1.37-1.58)
Current smoker	302	4.92	2.24 (1.97-2.55)
**Peripheral arterial disease**			
Never smoker	887	1.06	1.00
Past smoker	991	1.87	1.77 (1.61-1.95)
Current smoker	384	4.80	5.03 (4.44-5.71)
**Other major CVD**			
Never smoker	7944	9.58	1.00
Past smoker	5276	10.57	1.10 (1.06-1.14)
Current smoker	948	11.91	1.24 (1.15-1.32)
**ANY MAJOR CVD**			
**Never smoker**	14442	18.70	1.00
**Past smoker**	10082	21.38	1.16 (1.13-1.19)
**Current smoker**	2247	29.05	1.60 (1.53-1.68)

*Age-sex standardised rate per 1000 person-years.
RR (relative risk) adjusted for age, sex, region of residence, alcohol consumption, annual household income, and education attainment; adjustment for sex is through stratification for outcomes IHD (stratified for education as well), AMI, non-AMI, IHD, heart failure (stratified for income as well) and major CVD. For definitions of outcomes see Additional file 2: Table S1.

Table S5. CVD subtypes (level 3 ICD-10 AM codes) ranked according to frequency of fatal and non-fatal outcomes

**Non-fatal outcomes**		
ICD-10-AM codes	Number	%*
I48 Atrial fibrillation and flutter	8537	31.9
I25 Chronic ischaemic heart disease	8442	31.5
I21 Acute myocardial infarction	4216	15.7
I20 Angina pectoris	4212	15.7
I50 Heart failure	3805	14.2
G45 Transient cerebral ischaemic attacks and related syndromes	1989	7.4
I80 Phlebitis and thrombophlebitis	1860	6.9
I63 Cerebral infarction	1709	6.4
I26 Pulmonary embolism	1629	6.1
I47 Paroxysmal tachycardia	1588	5.9
I44 Atrioventricular and left bundle-branch block	1537	5.7
I49 Other cardiac arrhythmias	1532	5.7
I70 Atherosclerosis	1299	4.9
I35 Nonrheumatic aortic valve disorders	1108	4.1
I51 Complications and ill-defined descriptions of heart disease	968	3.6
I64 Stroke, not specified as haemorrhage or infarction	845	3.2
I27 Other pulmonoary heart diseases	787	2.9
I42 Cardiomyopathy	665	2.5
I71 Aortic aneurysm and dissection	655	2.4
I34 Nonrheumatic mitral valve disorders	644	2.4
I65 Occlusion and stenosis of precerebral arteries, not resulting in cerebral infarction	575	2.1
I46 Cardiac arrest	551	2.1
I61 Intracerebral haemorrhage	513	1.9
I67 Other cerebrovascular diseases	414	1.5
I69 Sequelae of cerebrovascular disease	403	1.5
I62 Other nontraumatic intracranial haemorrhage	307	1.1
I72 Other aneurysm and dissection	300	1.1
I77 Other disorders of arteries and arterioles	287	1.1
I74 Arterial embolism and thrombosis	230	0.9
I12 Hypertensive kidney disease	122	0.5
I24 Other acute ischaemic heart diseases	122	0.5
I66 Occlusion and stenosis of cerebral arteries, not resulting in cerebral infarction	119	0.4
I73 Other peripheral vascular diseases	103	0.4
I11 Hypertensive heart disease	73	0.3
I23 Certain current complications following acute myocardial, infarction	33	0.1
I22 Subsequent myocardial infarction	26	<0.1
I13 Hypertensive heart and kidney disease	19	<0.1
I36 Nonrheumatic tricuspid valve disorders	17	<0.1
G46 Cardiac arrest	13	<0.1
I28 Other diseases of pulmonary vessels	6	<0.1
**Total number of people with non-fatal major CVD outcomes**	**26771**	

**Fatal outcomes**		
ICD-10-AM codes	Number	%*
I21 Acute myocardial infarction	558	22.0
I25 Chronic ischaemic heart disease	530	20.9
I64 Stroke, not specified as haemorrhage or infarction	297	11.7
I50 Heart failure	179	7.0
I69 Sequelae of cerebrovascular disease	118	4.6
I61 Intracerebral haemorrhage	103	4.1
I48 Atrial fibrillation and flutter	95	3.7
I71 Aortic aneurysm and dissection	91	3.6
I62 Other nontraumatic intracranial haemorrhage	76	3.0
I63 Cerebral infarction	73	2.9
I35 Nonrheumatic aortic valve disorders	65	2.6
I11 Hypertensive heart disease	48	1.9
I51 Complications and ill-defined descriptions of heart disease	32	1.3
I26 Pulmonary embolism	31	1.2
I67 Other cerebrovascular diseases	30	1.2
I12 Hypertensive kidney disease	29	1.1
I73 Other peripheral vascular diseases	29	1.1
I42 Cardiomyopathy	27	1.1
I46 Cardiac arrest	21	0.8
I49 Other cardiac arrhythmias	19	0.7
I27 Other pulmonary heart diseases	16	0.6
I24 Other acute ischaemic heart diseases	13	0.5
I34 Nonrheumatic mitral valve disorders	13	0.5
I80 Phlebitis and thrombophlebitis	12	0.5
I70 Atherosclerosis	9	0.4
I77 Other disorders of arteries and arterioles	8	0.3
I13 Hypertensive heart and kidney disease	7	0.3
I72 Other aneurysm and dissection	<5	<0.2
I74 Arterial embolism and thrombosis	<5	<0.2
I44 Atrioventricular and left bundle-branch block	<5	<0.2
G45 Transient cerebral ischaemic attacks and related syndromes	<5	<0.2
I47 Paroxysmal tachycardia	<5	<0.2
**Total number of people with fatal major CVD outcomes**	**2540**	

*Percentages are the proportion of people with either non-fatal or fatal outcomes experiencing the specific CVD subtype. Percentages of non-fatal outcomes do not add up to 100% because they are not mutually exclusive.
I10 [Essential (primary) hypertension] and K55 [Vascular disorders of intestine] are not included in this table as they are not part of “any major CVD”. Counting these in (combining major CVD, I10 and K55), there were 34,791 people with non-fatal outcomes including 19,114 hospitalisations for I10 and 641 for K55. Similarly, there were 2,610 people with fatal outcomes including 44 deaths from I10 and 26 from K55.

Table S6: Outcomes for specific CVD subtypes (level 3 ICD-10-AM codes) with at least 50 events, in current and past smokers versus never smokers

A. Combined fatal and non-fatal outcomes

ICD-10-AM codes	Events			Std rate*			Current vs Never	Past vs Never
	Current	Past	Never	Current	Past	Never	RR (95% CI)	RR (95% CI)
I70 Atherosclerosis	253	587	465	3.00	1.10	0.56	6.60 (5.61-7.76)	2.02 (1.78-2.30)
I71 Aortic aneurysm and dissection	122	320	240	1.74	0.55	0.29	5.92 (4.71-7.45)	1.91 (1.60-2.27)
I74 Arterial embolism and thrombosis	41	93	99	0.40	0.17	0.12	4.12 (2.80-6.05)	1.46 (1.08-1.96)
I65 Occlusion and stenosis of precerebral arteries, not resulting in cerebral infarction	84	259	232	1.00	0.45	0.28	3.76 (2.90-4.88)	1.54 (1.28-1.86)
I72 Other aneurysm and dissection	45	120	138	0.47	0.25	0.16	3.25 (2.29-4.63)	1.41 (1.10-1.83)
I69 Sequelae of cerebrovascular disease	48	196	264	0.73	0.37	0.31	2.76 (2.01-3.81)	1.25 (1.03-1.53)
I67 Other cerebrovascular diseases	51	165	223	0.59	0.32	0.26	2.69 (1.96-3.70)	1.36 (1.10-1.68)
I73 Other peripheral vascular diseases	14	48	70	0.24	0.10	0.08	2.55 (1.40-4.64)	1.19 (0.81-1.75)
I21 Acute myocardial infarction	543	1687	2318	6.20	3.22	2.84	2.45 (2.22-2.70)	1.21 (1.13-1.29)
I27 Other pulmonary heart diseases	72	342	378	1.05	0.69	0.46	2.43 (1.87-3.16)	1.61 (1.37-1.87)
I46 Cardiac arrest	69	210	288	0.80	0.37	0.35	2.39 (1.81-3.15)	1.12 (0.93-1.35)
I24 Other acute ischaemic heart diseases	18	51	66	0.20	0.10	0.08	2.33 (1.35-4.01)	1.17 (0.80-1.72)
I50 Heart failure	305	1587	1982	5.05	3.24	2.41	2.23 (1.96-2.53)	1.46 (1.36-1.57)
I77 Other disorders of arteries and arterioles	31	120	140	0.35	0.21	0.15	2.16 (1.44-3.23)	1.50 (1.16-1.94)
K55 Vascular disorders of intestine	62	261	325	0.79	0.56	0.37	2.07 (1.56-2.74)	1.42 (1.19-1.68)
I63 Cerebral infarction	157	630	944	1.89	1.17	1.14	2.01 (1.69-2.40)	1.02 (0.92-1.14)
I42 Cardiomyopathy	78	255	348	0.75	0.45	0.40	1.88 (1.46-2.43)	1.10 (0.93-1.30)
I62 Other nontraumatic intracranial haemorrhage	27	145	190	0.30	0.27	0.22	1.87 (1.24-2.84)	1.20 (0.95-1.50)
I61 Intracerebral haemorrhage	45	185	311	0.56	0.36	0.36	1.84 (1.33-2.54)	0.96 (0.79-1.17)
I64 Stroke, not specified as haemorrhage or infarction	75	388	601	1.08	0.76	0.73	1.70 (1.33-2.18)	1.05 (0.91-1.20)
I12 Hypertensive kidney disease	10	51	85	0.22	0.09	0.10	1.63 (0.83-3.20)	1.06 (0.73-1.53)
I51 Complications and ill-defined descriptions of heart disease	85	391	523	1.00	0.69	0.61	1.62 (1.28-2.06)	1.12 (0.98-1.28)
I25 Chronic ischaemic heart disease	785	3497	4541	8.01	6.30	5.53	1.60 (1.48-1.73)	1.16 (1.11-1.22)
I11 Hypertensive heart disease	9	43	69	0.16	0.08	0.08	1.59 (0.77-3.26)	1.10 (0.73-1.64)
I47 Paroxysmal tachycardia	136	601	851	1.58	1.13	0.97	1.50 (1.24-1.80)	1.15 (1.03-1.29)
I26 Pulmonary embolism	132	581	928	1.51	1.09	1.04	1.41 (1.17-1.70)	1.09 (0.98-1.22)
I20 Angina pectoris	346	1612	2254	3.41	2.93	2.63	1.36 (1.21-1.52)	1.13 (1.06-1.21)
I10 Essential (primary) hypertension	1445	7234	10465	17.72	15.32	13.11	1.34 (1.27-1.42)	1.19 (1.16-1.23)
I48 Atrial fibrillation and flutter	538	3318	4700	7.41	6.45	5.69	1.31 (1.20-1.44)	1.13 (1.08-1.18)
G45 Transient cerebral ischaemic attacks and related	133	727	1130	1.88	1.45	1.28	1.31 (1.09-1.58)	1.09 (0.99-1.20)
I66 Occlusion and stenosis of cerebral arteries, not resulting in cerebral infarction	7	42	70	0.12	0.06	0.09	1.28 (0.58-2.83)	0.87 (0.58-1.29)
I80 Phlebitis and thrombophlebitis	136	690	1042	1.51	1.33	1.18	1.28 (1.07-1.54)	1.14 (1.03-1.26)
I35 Nonrheumatic aortic valve disorders	63	452	615	0.87	0.81	0.75	1.25 (0.96-1.63)	1.07 (0.94-1.22)
I49 Other cardiac arrhythmias	97	574	873	1.07	1.01	1.01	1.19 (0.96-1.48)	0.99 (0.89-1.11)
I44 Atrioventricular and left bundle-branch block	78	560	899	1.07	1.01	1.08	1.03 (0.82-1.31)	0.93 (0.83-1.04)
I34 Nonrheumatic mitral valve disorders	32	237	382	0.31	0.45	0.45	0.83 (0.57-1.19)	0.94 (0.79-1.11)

*Age-sex standardised rate per 1000 person-years. RR (relative risk) adjusted for age, sex, region of residence, alcohol consumption, annual household income, and education attainment; adjustment for sex is through stratification for outcomes I21, I25, I48, I50, I10, I50 (stratified for income as well) and I10 (stratified for alcohol and income as well). I10 and K55 are not part of "any major CVD" but were included due to their relevance as outcomes related to any major CVD.

B. Fatal outcomes only

ICD-10-AM codes	Events			Std rate*			Current vs Never	Past vs Never
	Current	Past	Never	Current	Past	Never	RR (95% CI)	RR (95% CI)
I71 Aortic aneurysm and dissection	17	46	28	0.28	0.10	0.03	8.47 (4.46-16.12)	2.74 (1.66-4.51)
I62 Other nontraumatic intracranial haemorrhage	9	24	43	0.09	0.05	0.05	3.28 (1.55-6.96)	0.90 (0.53-1.52)
I25 Chronic ischaemic heart disease	59	206	265	0.86	0.38	0.32	3.22 (2.39-4.33)	1.26 (1.04-1.53)
I21 Acute myocardial infarction	51	201	306	0.73	0.40	0.37	2.79 (2.04-3.80)	1.16 (0.96-1.41)
I69 Sequelae of cerebrovascular disease	6	45	67	0.17	0.10	0.08	2.43 (1.04-5.71)	1.36 (0.91-2.05)
I61 Intracerebral haemorrhage	8	34	61	0.15	0.07	0.07	2.22 (1.04-4.76)	0.99 (0.64-1.56)
I63 Cerebral infarction	6	20	47	0.12	0.04	0.06	2.19 (0.91-5.26)	0.72 (0.41-1.25)
I50 Heart failure	8	68	103	0.25	0.13	0.12	1.79 (0.86-3.75)	1.38 (0.99-1.92)
I48 Atrial fibrillation and flutter	<5	34	57	0.14	0.08	0.07	1.79 (0.64-5.01)	1.20 (0.76-1.91)
I64 Stroke, not specified as haemorrhage or infarction	13	101	183	0.28	0.21	0.22	1.59 (0.90-2.81)	0.98 (0.75-1.27)
I35 Nonrheumatic aortic valve disorders	<5	24	38	0.06	0.04	0.05	1.53 (0.46-5.12)	0.98 (0.56-1.69)

*Age-sex standardised rate per 1000 person-years.
RR (relative risk) adjusted for age, sex, region of residence, alcohol consumption, annual household income, and education attainment.
I10 and K55 are not part of “any major CVD” but were included due to their relevance as outcomes related to any major CVD.

Table S7. Smoking attributable fractions for CVD mortality and hospitalisation for acute coronary syndrome in Australia, using 2004-5 smoking prevalence estimates

	Age (years)	Observed rate in Australia per 100,000	Prevalence of current smoking in Australia, p_{cs}	Prevalence of former smoking in Australia, p_{fs}	Relative risk for current smokers, RR_{cs} (95%CI)	Relative risk for former smokers, RR_{fs} (95% CI)	**Smoking Attributable Fraction**	Smoking attributable rate per 100,000	**Attributable events**
**CVD mortality**									
Males	45–54	63	26.7	37.3	3.47 (2.76-4.37)	1.28 (1.05-1.57)	**43.4**	27.5	**419**
	55–64	142	20.2	47.3	3.47 (2.76-4.37)	1.28 (1.05-1.57)	**38.8**	54.9	**715**
	65–74	368	11.3	59.9	3.47 (2.76-4.37)	1.28 (1.05-1.57)	**31.0**	113.9	**1048**
	≥75	2299	5.5	68.2	2.12 (1.70-2.63)	1.18 (1.06-1.30)	**15.3**	352.0	**2208**
Females	45–54	22	21.3	29.0	3.47 (2.76-4.37)	1.28 (1.05-1.57)	**37.8**	8.4	**131**
	55–64	52	14.2	30.0	3.47 (2.76-4.37)	1.28 (1.05-1.57)	**30.4**	15.8	**210**
	65–74	155	8.4	29.3	3.47 (2.76-4.37)	1.28 (1.05-1.57)	**22.5**	34.9	**329**
	≥75	2273	4.0	26.6	2.12 (1.70-2.63)	1.18 (1.06-1.30)	**8.4**	190.0	**1610**
Total							**15.6**		**6671**
**Fatal and non-fatal acute coronary syndrome (ACS)**									
Males	45–54	386	26.7	37.3	2.65 (2.36-2.97)	1.19 (1.08-1.30)	**33.8**	130.3	**1985**
	55–64	717	20.2	47.3	2.65 (2.36-2.97)	1.19 (1.08-1.30)	**29.7**	212.9	**2774**
	65–74	1127	11.3	59.9	2.65 (2.36-2.97)	1.19 (1.08-1.30)	**23.0**	259.7	**2389**
	75-84	1861	5.5	68.2	1.76 (1.42-2.19)	1.19 (1.08-1.31)	**14.6**	271.5	**1281**
	≥85	3315	5.5	68.2	1.76 (1.42-2.19)	1.19 (1.08-1.31)	**14.6**	483.7	**751**
Females	45–54	125	21.3	29.0	2.65 (2.36-2.97)	1.19 (1.08-1.30)	**28.9**	36.1	**562**
	55–64	246	14.2	30.0	2.65 (2.36-2.97)	1.19 (1.08-1.30)	**22.5**	55.4	**738**
	65–74	489	8.4	29.3	2.65 (2.36-2.97)	1.19 (1.08-1.30)	**16.2**	79.3	**748**
	75-84	1099	4.0	26.6	1.76 (1.42-2.19)	1.19 (1.08-1.31)	**7.5**	82.1	**464**
	≥85	2436	4.0	26.6	1.76 (1.42-2.19)	1.19 (1.08-1.31)	**7.5**	182.0	**513**
Total							**19.5**		**12206**

Smoking prevalence estimates are from the Australian Health Survey 2004-5.

Observed rates of CVD mortality are those for all circulatory diseases (rather than any major CVD) in the Australian population, obtained from: Australian Institute of Health and Welfare. General Record of Incidence of Mortality (GRIM) books, 2018.

Observed acute coronary syndrome hospitalisations are from national data on hospitalisations for acute myocardial infarction (AMI) or unstable angina, obtained from: Australian Institute of Health and Welfare. Australia's Health 2016. Canberra: AIHW, 2016.

RR_{cs} and RR_{fs} used for CVD mortality are the broad age-group specific relative risks for mortality from major CVD among current and former smokers, respectively, relative to never smokers.

RR_{cs} and RR_{fs} used for acute coronary syndrome are the broad age-group specific relative risks for fatal and non-fatal AMI among current and former smokers, respectively, relative to never smokers.

Table S8. Selected grouped CVD subtypes in relation to smoking status at baseline by sex

A. Combined fatal and non-fatal outcomes

	**Men**			**Women**			
	Events	Std rate*	RR (95% CI)	Events	Std rate*	RR (95% CI)	$p_{interaction}$
**Ischaemic heart disease (IHD)**							
Never smoker	3284	10.28	1.00	2908	5.25	1.00	
Past smoker	3313	11.55	1.15 (1.09-1.21)	1208	5.87	1.18 (1.10-1.26)	0.02
Current smoker	691	14.83	1.56 (1.43-1.70)	374	9.02	1.86 (1.66-2.07)	
**Acute myocardial infarction (AMI)**							
Never smoker	1231	3.87	1.00	1087	1.96	1.00	
Past smoker	1244	4.30	1.15 (1.06-1.25)	443	2.29	1.31 (1.16-1.46)	0.02
Current smoker	365	7.98	2.26 (2.00-2.55)	178	4.61	2.87 (2.43-3.39)	
**IHD, excluding AMI**							
Never smoker	2942	9.02	1.00	2428	4.33	1.00	
Past smoker	2956	10.18	1.15 (1.09-1.21)	1034	4.89	1.17 (1.09-1.26)	0.04
Current smoker	606	12.45	1.49 (1.36-1.63)	312	6.91	1.76 (1.56-1.99)	
**Cerebrovascular disease**							
Never smoker	951	3.17	1.00	1182	2.17	1.00	
Past smoker	1048	3.53	1.14 (1.04-1.25)	439	2.31	1.12 (1.00-1.25)	0.9
Current smoker	231	6.15	2.20 (1.89-2.55)	140	4.00	2.12 (1.77-2.54)	
**Heart failure**							
Never smoker	781	2.71	1.00	1201	2.21	1.00	
Past smoker	1089	3.74	1.44 (1.31-1.59)	498	2.85	1.50 (1.34-1.67)	0.9
Current smoker	186	5.34	2.16 (1.83-2.55)	119	4.67	2.33 (1.92-2.83)	
**Peripheral arterial disease⍰**							
Never smoker	432	1.39	1.00	472	0.85	1.00	
Past smoker	730	2.50	1.79 (1.58-2.02)	285	1.47	1.84 (1.58-2.14)	0.9
Current smoker	254	5.88	5.07 (4.32-5.96)	138	4.02	5.17 (4.23-6.31)	
**Other major CVD**							
Never smoker	3448	11.19	1.00	4630	8.59	1.00	
Past smoker	3476	12.27	1.08 (1.03-1.14)	1878	9.47	1.13 (1.07-1.19)	0.8
Current smoker	562	14.16	1.24 (1.13-1.36)	406	10.78	1.26 (1.14-1.40)	
**ANY MAJOR CVD**							
**Never smoker**	6790	23.57	1.00	8038	15.72	1.00	
**Past smoker**	7027	27.10	1.15 (1.12-1.20)	3325	17.79	1.18 (1.13-1.23)	0.9
**Current smoker**	1437	37.47	1.63 (1.54-1.73)	894	24.57	1.64 (1.53-1.76)	

*Age standardised rate per 1000 person-years.
RR (relative risk) adjusted for age, region of residence, alcohol consumption, annual household income, and education attainment; for the heart failure outcome adjustment for income is through stratification, in both men and women. For definitions of outcomes see Additional file 2: Table S1.

B. Fatal events only

	Men			Women			
	Events	Std rate*	RR (95% CI)	Events	Std rate*	RR (95% CI)	$p_{interaction}$
**Ischaemic heart disease (IHD)**							
Never smoker	268	0.90	1.00	306	0.54	1.00	
Past smoker	314	1.06	1.23 (1.04-1.46)	100	0.62	1.30 (1.03-1.64)	0.7
Current smoker	76	2.34	2.91 (2.23-3.80)	37	1.14	3.40 (2.38-4.85)	
**Acute myocardial infarction (AMI)**							
Never smoker	132	0.46	1.00	174	0.31	1.00	
Past smoker	145	0.49	1.15 (0.90-1.47)	56	0.34	1.26 (0.92-1.72)	0.8
Current smoker	34	1.01	2.77 (1.87-4.11)	17	0.52	2.77 (1.66-4.64)	
**IHD, excluding AMI**							
Never smoker	136	0.44	1.00	132	0.23	1.00	
Past smoker	169	0.57	1.31 (1.04-1.65)	44	0.28	1.36 (0.95-1.94)	0.8
Current smoker	42	1.33	3.03 (2.11-4.35)	20	0.62	4.22 (2.56-6.95)	
**Cerebrovascular disease**							
Never smoker	161	0.58	1.00	252	0.46	1.00	
Past smoker	172	0.60	1.15 (0.92-1.43)	67	0.42	0.91 (0.69-1.21)	0.7
Current smoker	28	1.16	2.46 (1.63-3.71)	17	0.74	2.00 (1.21-3.30)	
**ANY MAJOR CVD**							
**Never smoker**	591	2.04	1.00	798	1.42	1.00	
**Past smoker**	688	2.34	1.23 (1.10-1.38)	245	1.51	1.15 (1.00-1.34)	0.6
**Current smoker**	139	4.66	2.67 (2.21-3.24)	79	3.12	2.90 (2.28-3.68)	

*Age standardised rate per 1000 person-years.
RR (relative risk) adjusted for age, region of residence, alcohol consumption, annual household income, and education attainment. For definitions of outcomes see Additional file 2: Table S1.
